# Assessing the Role of Experimental Evidence for *Interface* Judgment: Licensing of Negative Polarity Items, Scalar Readings, and Focus

**DOI:** 10.3389/fpsyg.2018.00059

**Published:** 2018-02-21

**Authors:** Anastasia Giannakidou, Urtzi Etxeberria

**Affiliations:** ^1^Department of Linguistics, University of Chicago, Chicago, IL, United States; ^2^Centre National de la Recherche Scientifique, IKER (UMR 5478), Bayonne, France

**Keywords:** interface judgment, negative polarity items, scalar items, FOCUS, prosody

## Abstract

This paper reviews a series of experimental studies that address what we call “interface judgment,” which is the complex judgment involving integration from multiple levels of grammatical representation such as the syntax-semantics and prosody-semantics interface. We first discuss the results from the ERP literature connected to NPI licensing in different languages, paying particular attention to the N400 and the P600 as neural correlates of this specific phenomenon and focusing on the study by Xiang et al. (2016). The results of this study show evidence that there are two distinct NPI licensing mechanisms, i.e., licensing and rescuing, in line with Giannakidou (1998, 2006). Then we discuss an acceptability judgment task on Greek NPIs which supports the negativity as a scale hypothesis (Zwarts, 1995, 1996; Giannakidou, 1998). For the semantics-prosody interface judgment, we discuss two types of findings on two different phenomena and languages: (i) the study by Giannakidou and Yoon (2016) on scalar and non-scalar NPIs in Greek and Korean, which serves as the foundation for Chatzikonstantinou's (2016) study of production data showing distinct prosodic properties in emphatic (scalar) and non-emphatic (non-scalar) Greek NPIs; (ii) a (production and perception) study by Etxeberria and Irurtzun (2015) on the prosodic disambiguation of the scalar/non-scalar readings of sentences containing the focus particle “ere” in Basque. The main conclusion of the paper is that experimental methods of the kind discussed in the paper are useful in establishing physical, quantitative correlates of interface judgment.

## Framing the topic: meaning and interface judgment

What does it mean for speakers to have a linguistic judgment about meaning? How does the semantic judgment differ from the judgment about syntax, and how are the two to be distinguished from use errors or lexical failures? Are there hallmarks of syntax-semantics integration, and if so what are they? Are there grammatical phenomena that allow us to pinpoint physical correlates of this integration? Since sentences are typically *uttered*, what role does prosody play in disambiguating or bringing about additional dimensions of meaning that reflect different modules (semantics vs. pragmatics)? These are some of the questions that we discuss in the present article.

Let us define “interface judgment” as the judgment that comes from integrating representations from multiple grammatical levels such as, for instance, syntax and semantics, and semantics and prosody. The issues of interface judgment have *not* featured prominently in the older experimental literature, which tended to mostly study the nature of morpho-syntactic judgment. The reason is understandable: morphological and syntactic success or failure, such as e.g., person, gender, number agreement, or grammatical case inform us directly about what is generated (or not) by the grammar, and the judgment seems pretty robust. Since Chomsky (1957, 1964), the field has generally accepted that speakers' reactions to, and intuitions about, “grammatical” and “ungrammatical” are relatively clear. Grammatical is a structure that is generated by the rules of the grammar; ungrammatical is one that cannot be generated by the grammar. Chomsky notes that speakers' intuitions about grammaticality differ from intuitions about mere anomaly. Notice the contrast below:

(1) Apples grow on trees (grammatical, meaningful).
(2) ^*^Apples growed on trees (ungrammatical: ill-formed morphology of past tense).
(3) #Apples grow on noses (grammatical, semantically anomalous).

Semantic anomaly is distinct from morphosyntactic failure (^*^) and is marked with #, as indicated in (3). In (3), anomaly arises because of *lexical incompatibility*: the predicate *grow* doesn't combine conceptually with “on noses” when it comes to apples; apples don't grow on noses but on trees, in the US, in the fall, and the like. Warts, on the other hand, may indeed grow on noses. Likewise, (4) is odd because *Jason* is the name of a person, and a person cannot combine with the predicate “has a population of 3 million.”

(4) Athens has a population of 3 million.
(5) #Jason has a population of 3 million.

(4) is semantically well-formed while (5) is ill-formed due to the anomalous combination of subject and predicate. Sentences like (5) are known as category errors. We are dealing clearly with lexical mismatches that are nevertheless grammatical, i.e., generated by the grammar.

Other cases of semantic anomaly are produced with tautology and contradiction:

(6) #Daniel Day-Lewis is Daniel Day-Lewis.
(7) #It is raining outside and it is not raining outside.

These sentences are under-informative and therefore meaningless. A tautology is always true (analytic), and a contradictory sentence is always false; neither sentence, therefore, conveys information. Uninformative sentences are *infelicitous*—and often speakers try to make sense by enriching the meaning. For instance, (6) can be used to mean that Daniel Day-Lewis is a great actor, so even in a bad movie he will shine. Likewise (7) can be manipulated to mean that it is not *truly* raining, i.e., it rains only a little bit. Speaker meaning is malleable this way as hearers strive to make sense of the infelicitous messages they get^1^.

The question is: is semantic judgment a felicity judgment, or is it more complex including interaction with morphosyntax, and perhaps prosody? This is not a trivial question to ask. Indeed, there are linguistic phenomena suggesting that semantic judgment is complex, and relies on *integrating* information from multiple levels of grammatical representation such as semantics, morphosyntax, and prosody. These three are central levels of grammatical representation, and studying their interfaces can be very useful in uncovering the nature of complex linguistic judgment. Two phenomena stand out as particularly illustrative cases: anaphoric pronouns and negative polarity items (NPIs), and we will study the latter here. Both involve syntax-semantics long distance dependencies in that the distribution of the anaphor and NPI are constrained because of a semantic requirement that forces a particular syntactic relation that is not local (i.e., it does not involve adjacent elements). It is therefore no surprise that there have been attempts to unify the two phenomena (Progovac, 1994). Giannakidou's (1998) and Giannakidou and Quer's (2013) concept of *dependent variable* (to be discussed later) can be understood as the semantic correlate of a syntactic anaphor.

The early literature (Ladusaw, 1980) defended a syntax-semantics view of NPIs as ungrammatical due to semantic and syntactic constraints; more recently, Giannakidou (1997, 1998), and Giannakidou and Quer (2013) develop this integration view further. Some literature, however, obliterates the difference between felicity and grammaticality, and treats NPI failures on a par with infelicities. This path had been taken in the early pragmatic scales tradition (Fauconnier, 1975; Israel, 1996, 2011), and has recently been pursued by Kadmon and Landman (1993), Krifka (1995), and to a certain extent by Chierchia (2006, 2013). In these approaches, NPI failures are characterized as “severe” infelicities, or “special” contradictions that lead to ungrammaticality (see also the free choice accounts in this spirit such as Aloni, 2007; Menéndez-Benito, 2010). The deciding criterion is not speaker's intuition or a concrete psycholinguistic measure; typically, no deciding criterion is offered. Sometimes, authors refer to a post-compositional computation (suggested by Gajewski, 2002 in unpublished work) as a recipe for when to call a contradiction “grammatical,” and when to call it “ungrammatical.” However, unless we have some appeal to intuition or some other kind of evidence, it is difficult to see the distinction between a “grammatical” and “ungrammatical” contradiction as more than just highly speculative—a mere declaration, in fact, of the choice to avoid addressing the complexity in the nature of NPI licensing.

NPIs are words like *any, either, ever* which systematically fail when they do not occur in the scope of negation:

(8) a. ^*^Bill brought any presents.
b. Bill didn't buy any presents.
c. ^*^Bill talked to John either.
d. Bill didn't talk to John either.

Negation therefore “licenses” the NPI. The early literature (Klima, 1964; Ladusaw, 1980; Linebarger, 1987) typically marks NPI failures with ^*^ and not #, reflecting the judgment that the NPI failure is not a mere use-based failure or lexical anomaly. Ladusaw proposed the concept of *semantic filtering*. Semantic filtering requires a module of grammar, just like binding theory (Chomsky, 1981), and this is a clear admission that NPI licensing is a grammatical and not a merely pragmatic phenomenon. The reason why NPIs fail is that they need to co-occur with a licenser, and the licenser has a specific semantic property. NPI-licensing, then, illustrates a synergy between semantics and syntax, and raises the question of well-formedness that is determined by both. In addition to *any, either*, we see indeed that many NPIs appear to be subject to severe grammatical constraints, e.g., Greek NPIs, “n-words” (see Laka, 1994; Giannakidou, 2006; Giannakidou and Zeijlstra, 2017). Generally, the literature on NPIs (which we will not review here, but see Giannakidou, 2011 for a recent overview) has shown that a substantial part of NPI violations involve grammatical violations that have to do with syntactic constraints too. Corpus studies such as Hoeksema (2010) on Dutch and English NPIs have also been instrumental in illustrating the (often severely) limited distributions of NPIs.

So, how does this semantico-syntactic integration judgment differ from infelicity, i.e., the merely pragmatic conflict of lexical anomaly and uninformativity? It is important to raise this question because sometimes, as we mentioned earlier, the difference is blurred. Based on intuition alone it is hard to make the case for different *types* of judgment—and here the contribution of experiments and quantitative data can be instrumental, we will argue.

In what follows, we will review literature that reveals the physical (neural) correlates of the “interface judgment,” which we take to be the judgment typical of interface phenomena that rely on integrating multiple levels of grammatical representation. We will study two prominent cases of interface judgment involving syntax-semantics and prosody-semantics integration. We will review (a) ERP literature showing distinct neural patterns for syntax-semantics integration with NPIs, (b) experiments, including mere acceptability judgment tasks, illustrating the usefulness of such methodology in extracting more refined sets of data, and (c) interaction of prosody with scalar structure and focus. Regarding focus, we will study recent work on the Basque focus particle *ere* “also/even.” Our goal is to show that experimental methodologies can be instrumental in revealing richer sets of data that make visible interactions between levels of grammatical representation—one can therefore be hopeful that experimental methodologies, at least of the kind discussed here, can further aid the understanding of syntax-semantics and prosody-semantics-pragmatics interface.

The promise, of course, does not come without open questions, especially since this is a new research paradigm with various methodologies. There certainly are many questions to be explored in terms of comparing the methodologies. However, it is impossible to offer a general comparison between methodologies in this short survey; our focus is rather the empirical phenomena of NPI-licensing, focus, and the particle *even*.

One might think that perhaps the notion of integration judgment is too broad, and that upon reflection it might be hard to think of language phenomena that do not, to some extent, require reference to multiple levels or interfaces (We thank John Drury for raising this point). While this concern is well taken, we believe it is overstated. There are plenty of morphosyntactic phenomena that do not make recourse to additional grammatical levels. For example, a failure to indicate the correct morphological tense in our earlier sentence (2) (^*^growed, instead of *grew*) does not require integration; nor do case assignment, and various kinds of agreement. For such phenomena, it is accepted that we don't need a level other than morphosyntactic computation, and we are not aware of any analyses that treat these phenomena as involving (non-trivial) integration. At the same time, it is true that integration phenomena are plentiful. If we can convince that experimental methodologies of the kind discussed here are helpful in teasing apart the various grammatical levels in NPI licensing and with focus, then we can be optimistic that such methodologies carry promise for other integration phenomena too^2^.

The paper develops as follows. In section NPI Licensing and the Syntax-Semantics Interface, we start with discussion of the interface nature of NPI licensing. We conclude that NPI licensing is a dependency that involves both semantics (matching, binding) and syntax (c-command). In section Interface Judgment with NPIs: Two Recurring Components, we discuss ERP evidence for the two components. In section Empirical Variation and the Scale of Negativity, we discuss an acceptability judgment study revealing a gradient status for NPI licensers in terms of negativity in Greek. In the first part of section Intonation and Meaning: Disambiguating Scalar Components, we discuss the role of intonation in Greek NPIs relying on recent work by Chatzikonstantinou (2016), who presents evidence that prosodic prominence in Greek NPIs indicates the presence of a scalar component. Finally, in the second part of section Intonation and Meaning: Disambiguating Scalar Components, we discuss the Basque additive particle *ere* “also/even,” shown in Etxeberria and Irurtzun (2015) to obtain scalar readings with prosodic prominence. We conclude in section General Conclusions.

## NPI licensing and the syntax-semantics interface

Polarity is a pervasive phenomenon in natural language, and has received considerable attention since Klima's (1964) seminal work on English negation (see Giannakidou, 2011, 2017 for overviews and references). A “negative polarity item” (NPI), as we said earlier, is an expression that has limited distribution because it requires a preceding negation. Recall:

(9) Nicholas didn't say anything.
(10) ^*^Nicholas said anything.
(11) Nicholas hasn't ever talked to Ariadne.
(12) ^*^Nicholas has ever talked to Ariadne.

*Anything* and *ever* are NPIs and need negation for grammaticality^3^; negation is said to license the NPI, it is thus the “licenser.” An NPI-licenser can be non-negative but also nonveridical (Zwarts, 1995; Giannakidou, 1997, 1998 among others). NPIs therefore appear, in addition to negation, also in questions, with modal verbs, imperatives etc. We illustrate below with *any* and Greek NPIs:

(13) a.   Milises        me          kanenan? question
             talked.2sg   with        anybody
             Did you talk to anybody?
        b.  Patise          kanena   pliktro.   imperative
             press.imperany.NPI key
             Press any key.
        c.   O                Janis      bori        na                  milisi
             the               John      may        subj               talk.3sg
             me               kanenan       kathijiti (an theli)
             with             anybody       professor (if wants.3sg)
             John may talk to anybody (if he wants to).

**Figure 1:**
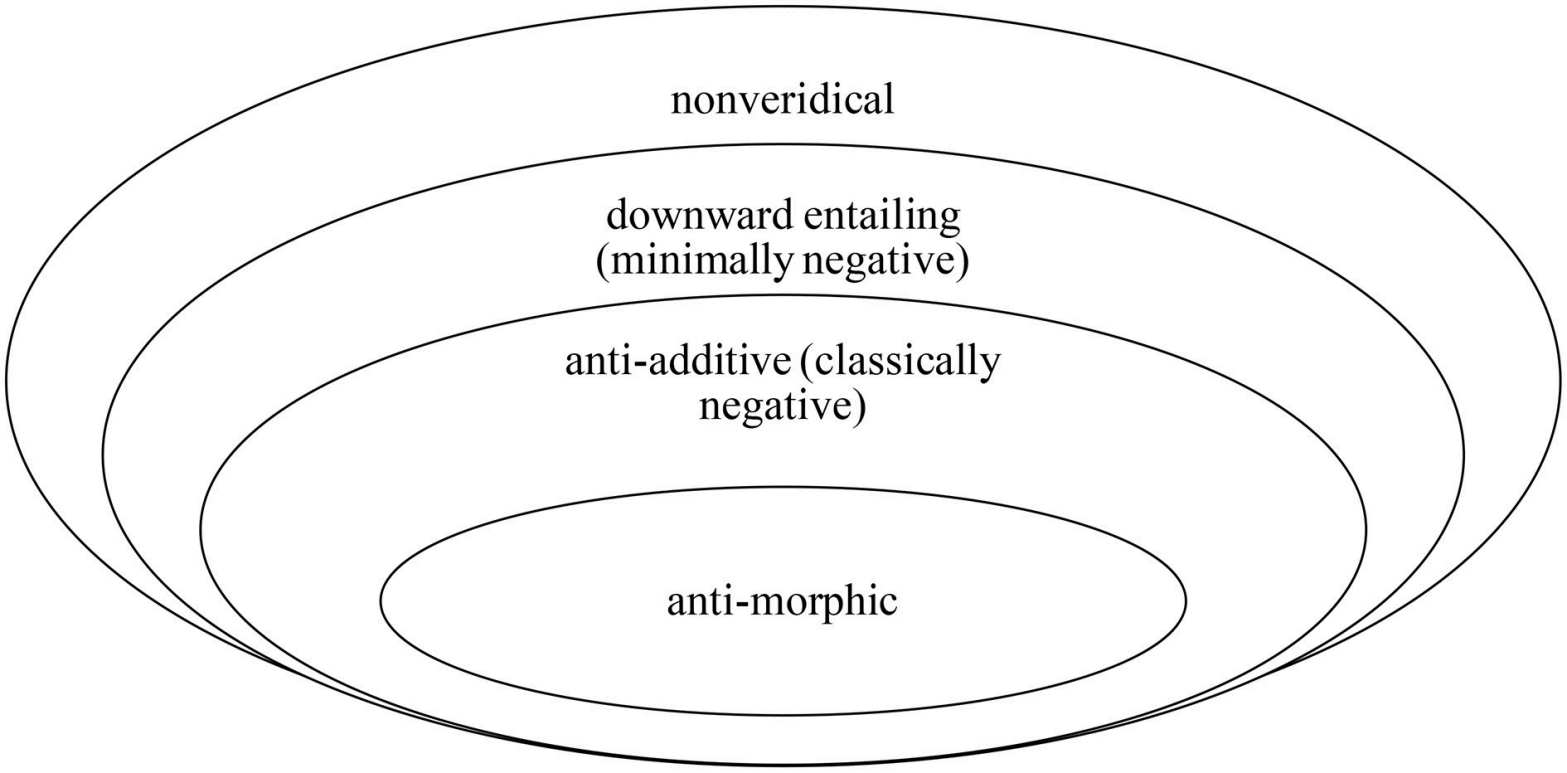
The Giannakidou/Zwarts Nonveridical Hierarchy of polarity contexts.

Questions and modals are nonveridical, i.e., they don't entail the truth of ϕ. Negation is also nonveridical: *not* ϕ does not entail ϕ. NPIs appear in nonveridical contexts, i.e., contexts in the scope of nonveridical operators. As summarized below, the negative is also nonveridical (Figure 1).

Nonveridicality includes negation as a subcase while allowing a wider distribution of NPIs in non-negative contexts. Downward entailment is the minimum of negativity (or, *minimal negation*, see Zwarts, 1995). Anti-additive and anti-morphic operators (*nobody, not*) are classically negative, which means they are stronger: they satisfy three (anti-additive) or all four (anti-morphic) of the de Morgan laws for complementation. Giannakidou and Zwarts, therefore, envision NPI licensers as having *gradient* strength: nonveridical non-negative licensers such as modals and questions are the weakest, negative licensers are stronger than non-negative, and within the negative class, minimal negation is weaker than classical negation. This predicts, as is easy to see, differences in the licensing potential of expressions, and in section Empirical Variation and the Scale of Negativity we show that a simple acceptability judgment task can detect empirically the differences^4^.

In the present paper, we focus on the interaction of NPIs with negation. NPIs need negation to be licensed. Licensing, crucially, is both a semantic and a syntactic requirement: it is the semantic requirement that there be a negation in the sentence, and the syntactic requirement that the NPI *be in the scope* of negation, which translates into a need for the NPI to be c-commanded by negation:

(14) a. Bill didn't see any student.
b. ^*^Any student didn't see Bill.
(15) a. ¬∃x. student (x) ∧ see (Bill, x)
b. ∃x. student (x) ∧ ¬ see (Bill, x)

*Any student* can only be interpreted in the scope of negation—the scoping in (15b) where there is one person such that Bill didn't see her (but others that he saw), is impossible. Furthermore, appearance of *any* to the left of negation is generally prohibited (14b) (though there are NPIs that are not subject to this surface constraint, e.g., Greek NPIs and the Turkish NPIs considered in Yanilmaz and Drury, 2018). In the example below, we see more effects of the syntactic constraint on c-command:

(16) ^*^The sister of no student said anything.
(17) No student said anything.

*No student* licenses *any* in (16) but not in (17) because, although *no student* linearly precedes the NPI, it does not c-command it. NPI licensing thus manifests a true semantics and syntax dependency, and presents a prime case for studying the interaction between these two levels. Giannakidou (1997) formulates licensing as follows:

(18) a. *Licensing* (Giannakidou, 1997)
b. R (β, α); where R is the scope relation; α is the polarity item; β is a negative or nonveridical expression which serves as the licenser.

Licensing requires that the NPI α be in the scope of β. R is a “scope” relation. Scope is both a semantic relation—a matching relation of polarity or sensitivity features (Giannakidou, 1997; Zeijlstra, 2004)—and the syntactic relation of c-command, or clause boundary (as it appears in Greek, Turkish, and Romance n-word NPIs)^5^.

Let us see now how NPI licensing is manifested in experimental data. Based on intuition alone, it is very hard to tease apart the semantic from the syntactic component of licensing.

## Interface judgment with NPIs: two recurring components

NPIs have been studied in the past 15 years with the aid of event-related potentials (ERPs). This technique has been useful because it examines the temporal dynamics of syntactic, lexical semantic, and potentially syntactico-semantic dimensions of language processing—and the data for NPIs have been relatively consistent. ERP-profiles with NPIs have systematically included both LAN/P600 and N400 effects. The P600 is linked to syntactic processing and more broadly integration processes (as we elaborate later, see Kuperberg, 2013); N400 can be taken to index lexical semantic processing, or semantic matching (as argued recently in Xiang et al., 2016), in a way consistent with the view that NPI-licensing involves semantico-syntactic integration.

### N-400 and lexical matching

As the name suggests, the N400 is a negative-going waveform that peaks at approximately 400 ms, with a primarily centro-posterior scalp distribution. The amplitude of the N400 evoked by an incoming word indexes the degree to which that word's semantic features match semantic features that have been pre-activated in the context at the time of encounter (Lau et al., 2008; Kutas and Federmeier, 2011; Kuperberg, 2013). The term “pre-activation” has often been associated with active prediction of specific lexical items, but Xiang et al. (2016) use it to refer more generally to the activation of relevant semantic features, regardless of whether active prediction or expectation of the upcoming word is at work.

Lau et al. (2008) offer a very lucid discussion of how the N400 can be used to reflect semantic effects related to “anomaly” and “expectation”—both relevant for NPI licensing as we saw at the beginning of the paper. The N400 response involves the presentation of a congruent or incongruent word before a word target (such as “coffee–tea” or “chair–tea”). A “semantically supportive” context elicits a response of smaller amplitude in the 300–500 ms interval, and although the effects of sentential context on the N400 response may be bigger in magnitude, collectively, we refer to this modulation as the “N400 effect.” Lau et al. state further that the amplitude of the N400 response “is modulated not only by the degree of anomaly *per se*, but also by *predictability*. [emphasis ours]. A *less expected* sentence endings generate a *larger* N400 [emphases ours] response than highly expected ones, even when both endings are semantically congruent (for example, “I like my coffee with cream and honey” would generate a larger N400 response than “I like my coffee with cream and sugar”) (Lau et al., 2008 p. 921).

In the context of NPI licensing, the (negative or nonveridical) licenser establishes an expectancy that, given the above, predicts an N400 response on the NPI. Expectancy and predictability explain why lexical anomalies (“I like my coffee with cream and sugar/socks,” “Apples grow on noses”) typically show the effect, but it would be erroneous to state that N400 indexes merely semantic anomaly (as it has indeed been stated in the past). The N400 is expected to show up in more patterns of incongruence, as indeed is the case (see Lau et al. for data and specific references). For NPIs, the presence of the N400 will be seen in this light, in particular indexing semantic matching. Following Giannakidou (1997) and Giannakidou and Quer (2013), it is reasonable to assume that the NPI contains a lexical polarity feature that marks it as a dependent item sensitive to negation. The following is a definition for NPI after Giannakidou and Quer (2013):

(19) *Negative Polarity items*
Denotation:             [[ NPI (*any, kanenas*)]] = P(x_*d*_); where *x*_*d*_
                              is a *dependent variable* in need of binding;
                              P stands for the NP predicate.
Licensing:               The NPI is an expression whose feature
                              structure contains an uninterpretable
                              POL(arity) feature whose value is
                              *nonveridical*, [*u*Pol:nonver]; this feature
                              must enter an Agree relation with a
                              [*i*Pol:nonver] head.

In the denotation, the NPI contains a *non-deictic* dependent variable in need of binding.

(20) *Non-deictic dependent variable* (Giannakidou, 2011)
A variable *v* is non-deictic iff *v* cannot be interpreted as a free variable.

We can also think of the dependent variable as a variable that cannot introduce a discourse referent (or, cannot be closed by text level existential closure, as suggested in Giannakidou, 1998). Such a variable won't be able to get a value from the context, unlike non-dependent variables that can, and will always appear to be “narrow scope”: its distribution will be constrained in contexts where there is an operator it can be bound by. The presence of a dependent variable thus underlies the very essence of NPI-hood.

The presence of a dependent variable creates limited distribution. The dependent variable class includes NPIs—but also non-polarity variables such as reflexive pronouns, traces, distributivity markers (reduplicated indefinites in Hungarian; Farkas, 1998), the temporal variable of the subjunctive mood (“temporal” polarity in Giannakidou, 2009), and as recently argued in Grano (2011), subjects of exhaustive control verbs such as *try, manage*, etc. This framework imposes an isomorphism between semantics (dependent variable that cannot remain free) and morphosyntax (a dependent variable being a distinct syntactic object from a non-dependent variable). Being a distinct syntactic object means, by *licensing*, that the NPI has a polarity feature. This polarity feature POL is subject to a matching requirement (*Agree*) with the licenser. In other words, the non-deictic variable dependency is lexically encoded in the POL feature. Polarity features have been implied for NPIs since the early days (e.g., Klima's +affective feature, Giannakidou, 1997 sensitivity features, Zeijlstra's NEG feature; Chierchia's +σ feature is within the same spirit)^6^.

With NPIs that are more narrowly sensitive to negation, such as e.g., n-words, or the Dutch NPI *hoeven “need,”* it is reasonable to assume that the abstract lexical semantic feature is not POL but [+Neg] (see Lin, 2015 for some recent discussion on the acquisition of this feature and its contrast with acquisition of broader NPIs such as any). In the literature on n-words it is very common to assume [+NEG] (Zeijlstra, 2004; Giannakidou and Zeijlstra, 2017 for an overview). In any case, POL and NEG would be the lexical indexing of the NPI-dependency in the grammatical representation of the NPI word. During the incremental comprehension of a sentence, if NEG or POL are compositionally derived prior to encountering the NPI (i.e., in the licenser), that should lead to a reduced N400 on the NPI. This hypothesis relies on the fact that the amplitude of the N400 evoked by an incoming word indexes the degree to which that word's semantic features match semantic features that have been preactivated by its context at the time of encounter (Lau et al., 2008; Kutas and Federmeier, 2011; Kuperberg, 2013). The term “pre-activation” has often been associated with active prediction of specific lexical items. (Xiang et al., 2016) use it in a more neutral sense to refer to the activation of relevant semantic features, regardless of whether active prediction or expectation of the upcoming word is at work, ahead of encountering the full linguistic input. In the context of NPI licensing, it is reasonable to assume that, during the incremental comprehension of a sentence, if a semantic [+NEG] feature or [+POL] feature is compositionally derived prior to encountering the NPI, that should lead to a reduced N400 on the NPI word.

In a series of studies (Saddy et al., 2004; Drenhaus et al., 2005, 2006, 2007), a reduced N400 with a central maximum was found on the German NPI *jemals* (“ever”) when it was licensed by negation, compared to the ungrammatical counterpart when *jemals* was not licensed. Similar N400 effects were also found for Dutch (Yurchenko et al., 2013) and English NPIs (Shao and Neville, 1998), a MEG study by Tesan et al. (2012), Xiang et al. (2009), and Xiang et al. (2016). The studies differed considerably in their aims, materials and experimental designs, behavioral task, and test language; but the N400 finding is in line with the idea that NPI licensing involves a semantically driven dependency and suggests that the semantic requirement of the NPIs involves some kind of lexical/morphological feature matching.

Interestingly, another study by Steinhauer et al. (2010) did not find an N400 difference between licensed and unlicensed *ever*^7^; but a crucial difference between their study and the others mentioned above is that Steinhauer et al. had a larger set of licensors in their stimuli, including various negative licensors such as *not, without, rarely*/*hardly*, and also licensers that are not negative *per se*, but non-veridical, such as *every, before, whether*, and yes-no questions. It is possible that negative features are only present with classically negative expressions (*no, no one, not*), and that the varying degree of negativity (or no negativity at all, e.g., with a non-veridical non-negative licenser) causes the N400 effect to be reduced. In Steinhauer et al. the effect could have been watered down by using both negative and non-negative licensors. This, by itself, of course raises the question of how to trace the judgment in the case of non-negative licensers, and this is something that needs to be studied. Overall, what we want to say here is that reduced N400 can be plausibly viewed as a correlate of the underlying lexical semantic matching dependency between the NPI and the licenser that produces an expectancy in the sense of Lau et al. (2008). The range of data confirming this is solid enough to be able to render the N400 effect a predictor. The weakened N400 with non-negative licensers observed in Steinhauer et al. may be a reflection of a matching between an NPI a non-negative licenser, either, as we said, because the non-negative licensers lack [NEG], or because it might have only [+POL]. If the NPI is [+NEG], but the licenser is only [+POL], this is a weaker match. In other words, there appears to be a hierarchy of strength of these lexical features.

At the same time, the presence of P600 effect with NPIs reflects syntactic integration, and the consistent presence of P600 allows us to treat these two components as tied to syntactico-semantic processing.

### Integration correlates and two modes of licensing

The majority of the NPI studies mentioned above also reported a posteriorly distributed P600 late positivity effect, which was larger for unlicensed NPIs than for licensed ones^8^. The P600 effect is typically associated with syntactic processing and syntactic complexity, and is reliably elicited by syntactic errors (Osterhout and Holcomb, 1992; Hagoort et al., 1993; see also Osterhout et al., 1994; Kaan et al., 2000; Phillips et al., 2005), or grammatical but syntactically complex constructions (Osterhout et al., 1994; Kaan et al., 2000; Phillips et al., 2005; Gouvêa et al., 2010). Although the precise functional interpretation of the P600 is yet to be determined, a broad generalization that has emerged is that it reflects costs associated with a processing stage in which information from different sources is integrated into one coherent representation. We will therefore take P600 to be an indicator of *integration* cost (Friederici and Weissenborn, 2007; Kuperberg, 2007; Bornkessel-Schlesewsky and Schlesewsky, 2008; Van Petten and Luka, 2012). Increased P600 amplitudes signal the detection of an integration error or difficulty^9^.

In the particular context of NPI licensing, multiple streams of information—syntactic, semantic, and in some cases pragmatic, as we mention next—are recruited to construct a grammatical representation that can license NPIs. In an ungrammatical sentence that does not license NPIs, the comprehension system fails to integrate an NPI into the current grammatical representation, and therefore produces a large P600^10^.

Crucially, sometimes NPIs are sanctioned pragmatically, by *implicit* negation. Giannakidou (2006) calls this phenomenon *rescuing* and it is very clear with emotive verbs:

(21) a. John was surprised that Mary has any friends.
b. We are lucky that we got any tickets!
c. John regrets that he talked to anybody.

In these sentences, *any* appears without a negative or nonveridical licenser; the emotive verb (*be surprised, be lucky, regret*), arguably, has a positive thus veridical presupposition that the complement is true. These are, at best, cases of mixed veridicality (Giannakidou and Mari, 2018), but some NPIs, as we see, appear nevertheless (see Baker, 1970; Linebarger, 1987 for earlier data). Giannakidou shows, on the other hand, that the corresponding Greek NPIs are ungrammatical:

(22) ^*^Metaniosa pou ipa tipota.
regret.1sg that said.1sg anything
I regret that I said anything.

As mixed licensers, emotive verbs are highly variable, as confirmed by a recent paper (Duffley and Larrivée, 2015) where it is shown that the appearance of *any* is indeed limited with emotives. Giannakidou argues that with emotives we don't have licensing proper but *rescuing* of the NPI by accessing implicit, i.e., not asserted, negation.

(23) *Rescuing by NEGATION* (Giannakidou, 2006).
A PI α can be rescued in sentence S, if the global context C of S makes a negative proposition S' available, and (b) α is in the scope of negation in S'.

Rescuing is proposed by Giannakidou a *secondary* mode of licensing that relies on pragmatic inferencing from the global context, which includes the presuppositions and implicatures of the sentence. Horn (2002) further structures global pragmatics with *assertoric inertia*: *one* component becomes assertorically inert, and another becomes salient. If the salient component contains negation, NPIs will be licensed. Emotive verbs give rise to a negative inference that has been characterized as an implicature (Linebarger, 1987), or presupposition (Baker, 1970; Giannakidou, 2006, 2016). In the case of emotives, then, the negative meaning arises not from a logical property of the emotive verb—which would render a NEG or POL feature possible—but from implicit negation (*regret* implicates or presupposes that *I wish I didn't*). With rescuing, the NPI needs to access the pragmatic level of representation.

In agreement with the rescuing idea, processing literature treats the appearance of NPIs with emotives as non-canonical, and uses labels such as *illusory* effect (Xiang et al., 2009, 2013), and *erroneous pragmatic licensing* (Yanilmaz and Drury, 2018). Giannakidou (1998) calls rescuing *indirect* licensing; Xiang et al. (2016) further study how licensing proper differs from non-canonical licensing in online computation. They look at the P600 effect. If the P600 indexes the integration effort with which an NPI is licensed, it provides a useful tool to examine whether or not negation in the pragmatics is treated by the comprehension system as an equally viable licenser.

Combining observations from the N400 and the P600 time windows, Xiang et al. state that they can construct a complete picture as to when and how negation is computed and used for grammatical purposes. The N400 reveals information about whether a negative meaning is established incrementally in context; the P600 assesses whether negative meaning, if available, can be immediately adopted to serve the grammatical function of NPI licensing.

Xiang et al. in their experiment 1 found the following. All (*no, few, only*) conditions that contain a legitimate licensor showed, expectedly, a qualitatively similar N400 reduction during the 300–400 ms time window at the critical NPI, relative to the unlicensed condition. They also by and large showed a reduced anterior negativity compared to the unlicensed condition during the late 700–900 ms time window. However, during the P600 time window, although conditions under *no, few*, and *only* showed qualitatively similar patterns involving a smaller P600 amplitude relative to the unlicensed condition, the emotive predicate condition *yielded a P600 as large as the P600 in the unlicensed condition*. This result sets rescuing with emotive predicates apart from licensing proper, and supports the thesis that rescuing does not involve syntax-semantic integration (as that would predict no P600). Trying to access negation in the pragmatics produces the effect. Giannakidou (1998, 2006) is the only currently available theory that predicts this fact, as it is the one that posits two qualitative distinct modes of licensing (licensing proper which involves syntax-semantics integration, vs. rescuing with involves pragmatics).

Let us note that Xiang et al. (2016) consider the possibility that the P600 effect may be due to fact that with emotives the NPI is embedded in a different clause, unlike in the other conditions where licenser and NPI are in the same clause (*No student said anything* vs. *Maria regrets that she said anything*). This is also a question raised by a reviewer. Xiang et al. therefore designed a second, self-paced reading experiment aiming to examine whether the ERP pattern could be replicated in a different paradigm, and also to assesses whether the additional processing cost found on the emotive condition is due to its NPI licensing properties or to the other possible sources of processing complexity (such as e.g., embedding). They conducted a 2 × 5-design self-paced reading study, the results of which rule out the possibility that the observed effects among the NPI conditions should be attributed to independent structural or contextual differences among different conditions. Of course, caution needs to be taken in drawing parallel relations between ERP and self-paced reading results; but the fact that the same costs, with the same relative timing, are observed in the NPI conditions from both the ERP data and the reading time data suggests that they are comparable measures to examine the online processing of NPI comprehension (see Xiang et al., 2016 for the experimental details).

What are the overall conclusions from this discussion? We suggest the following:

1. Experimental, specifically ERP, methodologies are useful in establishing physical, quantitative correlates of interface intuitions that can serve as criteria for distinguishing aspects of the linguistic judgment.
2. NPIs exhibit a complex judgment that involves integrations from multiple levels: syntax, semantics, and potentially pragmatics, as is in the case of rescuing.
3. Reduced N400 can be understood as the physical correlate of semantic licensing, i.e., as a matching relation between the NPI and its licenser.
4. P600 can be understood as the physical correlate of the syntactic aspect of licensing, i.e., integration.
5. The contrast between rescuing and licensing is real, and observed at the expected level of P600 as an integration effect.

Let us remind again that the N400 is not an index of felicity (as one would think, for instance by reading only, Shao and Neville, 1998), but of predictability, expectancy and matching. Overall, the ERP methodology allowed us to disentangle the key aspects of grammatical judgment with NPI licensing, in ways that would have been impossible with mere intuition. This, we believe, is a promising result—sufficient in itself to generate and enhance interest in pursuing these methodologies further for such types of phenomena.

Before we move on to different kinds of experiments, we want to offer a few comments on why, we think, ERP methodology is useful for NPI licensing, or, in other words, why tracking processing in time matters for this type of phenomenon. As it became clear, NPI licensing is a long-distance dependency: it requires ability of the processor to integrate material that may not be locally adjacent (*No* student saw *anything*). Other well-known long distance dependencies are wh-dependencies (*Who did Ariadne see t?*), and the antecedent—anaphor relations (*Ariadne likes talking to herself*) that we talked about at the beginning. In all these cases, e.g., upon encountering the NPI, the trace, or the anaphor, the processor must assess the structure that has already been processed. This raises the question of how structured representations are encoded in memory, and how representations are retrieved to extract information. Hierarchically structured representations must be tracked during language processing in order for the parser to “accurately single out grammatically licit antecedents, and representations of structure in memory must be organized in such a way that retrieval operations can make appropriate decisions about acceptable or unacceptable targets” (Xiang et al., 2009, p. 40). This entails that observing processing in time is an effective technique for assessing long-distance phenomena—and although the three phenomena mentioned here are distinct, they all involve explicit reference to previously processed lexical items and structure, hence they benefit from tracking processing on time. ERP methodology can thus provide a secure take, we believe, on this type of syntax-semantics integration.

We move on now to different methodology. We show that mere acceptability judgment tasks can also be useful in revealing more sharpened intuitions with NPIs.

## Empirical variation and the scale of negativity

NPIs, as mentioned earlier, are known to be licensed by classical and minimal negation, as well as nonveridical expressions that are not negative. NPI licensers can thus be viewed as being of variable strength when it comes to negativity, an idea expressed for the first time in Zwarts (1996). Recall the NPI licensers in Figure 1. Negation itself is the strongest licenser, but minimal negations (merely downward entailing such as *few*) are weaker, and non-negative licensers (questions, modals, etc.) are the weakest, with zero negativity. In the Zwarts and Giannakidou framework (Figure 1), negativity emerges as a gradient property, i.e., a scale:

(24) Scale of Negativity (Zwarts, 1996; Giannakidou, 1997)
<non-negative, mere downward entailment, antiadditive, antimorphic)>

*Nobody* is more negative than *few* (it satisfies three de Morgan laws, but *few* only satisfies two). And sentence negation is antimorphic, the strongest negation satisfying all de Morgan relations. Non-negative elements have zero negativity, which means that none of the negative laws apply. Chatzikontantinou et al. (2015) set out to test the predictions of this theoretical proposal. They also included the emotive verbs we discussed earlier and *only* which are of mixed veridicality.

We give below a brief description of their task. Seventy five native speakers of Greek in Greece were presented with 30 statements-pairs and were asked to point on a 1–5 scale whether the second statement is an acceptable continuation of the first. The second statement contained the NPI *pote* “ever”. The participants were asked to judge if the sentence is acceptable. Materials included five types of (S2) continuations differing on the (non)licensers. Sentence structure was kept as similar as possible e.g.,:

(25) (S1) Special effects are expensive.
(S2) {Elaxisti/ It surprised me that/Only} skinothetes
{Very few/It surprised me that/Only}directors
xrisimopiisan *pote* idhika efe.
used ever special effects.

Negative quantifiers are n-words in Greek that must co-occur with negation creating negative concord (mentioned earlier). This was the condition used for negation:

(26) Kanenas skinothetis dhen xrisimopiise *pote* idhika efe.
no director not used ever special effects
No director has ever used special effects.

It was expected that licensing proper would be the most solid judgment—and that, if Giannakidou and Zwarts' view of negativity is correct, we would have some variation in the data even with licensing. Overall the strength of licensers is:

(27) Negative strength of licensers: “>” indicates “stronger than”
negation > very few > only> factives > no-licenser

The results are the following:

**Table:** 

**Licenser**	**Dependency**	**Acceptability Rate**
sentential negation	Licensing	4.7
E$\lambda \overset{´}{\alpha }\chi \iota \sigma \tau$oι ‘very few’	Licensing, DE	3.5
μóνo ‘only’	Rescuing	2.5
emotive	Rescuing	2.3
no-licenser	–	0.3

These data show that each licenser was associated with different degree of acceptability, with all *t*-tests comparing conditions being highly significant. Chatzikonstantinou et al. ran a 1 way Anova and the results showed a main licenser effect (*F* = 121.337, *p* < 0001) which suggests that it matters what licenser you are. The analysis showed that apart from the comparison between *emotive factives* and *only* (*p* = 0.14) all other comparisons are statistically significant (*p* < 0.0001) which suggests a division among the NPI licensers.

These findings confirm the scalar negativity hypothesis. They indicate a distinction between licensing by negation and licensing by downward entailment (*elaxistoi* “very few”), and a difference between licensing proper [both cases in (a)] and the rescuing we discussed earlier. These differences cannot be captured in accounts that do not differentiate between modes of (licensing vs. rescuing; e.g., von Fintel, 1999) *and* strength of licensers. And, importantly, the variation in the data, again, could not have been revealed without the judgment task.

Hence, for interface phenomena such as NPI licensing, even simple quantitative methods can be helpful in revealing the empirical patterns that are relevant for theorizing. At the same time, as shown earlier, ERP methodology enables establishing physical, quantitative correlates of interface intuitions that can serve as criteria for distinguishing aspects of the linguistic judgment. NPIs exhibit a complex judgment that involves integrations from multiple levels: syntax, semantics, and potentially pragmatics, as is in the case of rescuing. Reduced N400 can be understood, we argued, as the physical correlate of the semantic aspect of licensing, i.e., as a matching relation between the NPI and its licenser, while P600 can be taken to index the syntactic aspect of licensing and the cost of integration.

## Intonation and meaning: disambiguating scalar components

We now want to study another kind of interface judgment: the one derived from prosody and semantics interface. Interactions between prosody and scope have been consistently noted in the literature on meaning and intonation, since they were first addressed in Jackendoff (1972). Actually, the question why and under which circumstances scope inversion is possible has provoked a fair amount of approaches, see references in Horn (1989: 226ff). Jackendoff (1972) noted that the example in (28), in an out-of-the-blue context is ambiguous between the interpretation in (28a) and (28b), depending on the scopal relation of the universal quantifier *all* and the sentential negation.

(28) All the men didn't go
∀ > ¬ : no man went
¬ > ∀ : some men went

As soon as intonation changes, this affects the sentence and only one of the readings is available. If the sentence is uttered with the rising contour, expressed by the lines below the example in (29a), the sentence is interpreted with negation taking scope over the universal quantifier *all*, i.e., *some men went*, while if the sentence ends with a falling pitch contour as in (29b), it is the universal quantifier that takes scope over the sentential negation and means that *no men went*. Büring (1997) and Krifka (1998) account for this data by making use of contrastive topicalization, that according to them, involves scope inversion in these cases. We will not get into discussing these proposals here, as we just want to show the facts.

(29) a. ALL the men didn't go. [B accent: ¬>∀]
\______________/
b. ALL the men didn't go. [A accent: ∀>¬]
\______________
           \

Other authors have also worked on the topic of the interaction of quantifier scope and prosody, e.g., Martí (2001), Kennelly (2003; 2004), Etxeberria and Irurtzun (2004), and Jackson (2007), etc.

In some other cases, prosody and information structure eliminate the ambiguity of a quantificational element. Thus, so-called weak Qs are assumed to be ambiguous between a cardinal and a proportional reading (cf. Milsark, 1977). Thus, a sentence like (30) exemplifies the two possible interpretations of *some* (cf. Partee, 1988).

(30) Some girls are playing basketball.

On its proportional interpretation, the meaning of *some* can be paraphrased as *some, but not others*, and it is synonymous with the partitive *some of the children*. This interpretation is felicitous only when the set of students is already under discussion. On its cardinal reading on the other hand, *ume batzuk* is not paraphrasable as a partitive, and the weak Q just makes reference to a quantity.

What appears to be extremely interesting is that as soon as prosody affects the sentence and the weak Q *some* is focalized, as exemplified in (31), the cardinal interpretation disappears and *some girls* can only be interpreted proportionally as *some, but not others*.

(31) [Some]_F_ girls are playing basketball.

Here again, as was the case in ambiguous sentences with two Qs, the focalization of the weak Q disambiguates the sentence.

We can find a similar effect with numerical noun phrases like three *beers*. The sentence in (32) is ambiguous between an *at least* interpretation and an *exactly* interpretation.

(32) I drank three beers.

Horn (1972) analyses the *at least* interpretation as an implicature, as shown by the felicity of (33) where the continuation eliminates the exactly interpretation of *three beers*.

(33) I drank three beers, in fact, I drank seven.

Crucially, as soon as the prosody of the sentence is changed by focalizing the numerical expression, we only get the *exactly* interpretation as shown by the ungrammaticality of (34).

(34) ^*^I drank [three]_F_ beers, in fact, I drank seven.

We get a similar effect here too, that is, as soon as we focalize the numerical expression one of the possible interpretations (the one we obtain via implicature, according to Horn, 1972) disappears.

The effect played by prosody has also been studied in other contexts such as answers to polarity questions (see Li et al., 2016, etc.), factive presupposition projections (Beaver, 2010; Tonhauser, 2016; Simons et al., 2017, etc.), etc. In this section we concentrate on scalar meanings that can be created by making use of prosodic marking. We first present production data from Chatzikonstantinou (2016) showing distinct prosodic properties correlating with scalar readings of Greek NPIs (5.1); then, we discuss a production and perception study by Etxeberria and Irurtzun (2015) on the prosodic disambiguation of the scalar/non-scalar readings of sentences containing the additive particle “ere” in Basque. In both cases we see prosody influencing or affecting semantic interpretation, and experimental methodology is, again, instrumental in refining and offering physical correlates of linguistic intuition. We will not discuss what explains the facts best, we will only concentrate on describing the facts.

### Greek NPIs

It has been a common observation that Greek exhibits a difference between two variants of NPIs distinguished by “emphatic accent” (Veloudis, 1982; Giannakidou, 1997 et seq., Tsimpli and Roussou, 1996); upper-case indicates the obligatory presence of prosodic prominence in a phrasal context. The emphatic NPI is interpreted as an n-word and participates in negative concord (Giannakidou, 1998, 2000), i.e., it requires negation and cannot appear in questions, unlike non-emphatic NPIs:

(35)     a.       kanenas/KANENAS     “anyone, anybody/no-one, nobody”
           b.       tipota/TIPOTA               “anything/nothing”
           c.       pote/POTE                      “ever/never”
           d.        puthena/PUTHENA        “anywhere/nowhere”
           e.      katholu/KATHOLU           “at all/not at all”
(36)    a.      Dhen idhe kanenan o Janis.
                    not saw NPI.person the John
                   “John didn't see anybody.”
           b.     ^*^Idhe kanenan/KANENAN o Janis.
                    saw.3sg NPI.person the John
                   Dhen idhe KANENAN o Janis.
                   not saw NPI.person the John
                 “John didn't see *anybody at all*.”
          d.     Idhes kanenan/^*^KANENAN?
                 Saw.2sg NPI.person
                Did you see anybody?
         e.     An dhis kanenan/^*^KANENAN
                 if see.2sg NPI.person
                 If you see anybody

Giannakidou and Yoon (2016) present a number of arguments showing that emphatic NPI gives rise to intensified, *scalar* negation, akin to *anybody at all* (38c), while the non-emphatic NPI (previously encountered) is non-scalar. To understand the contrast, consider the following scenario (from Giannakidou and Yoon, 2016):

(37) Context: Maria is supposed to read some articles this week for *Semantics 2*, of which only one is required (the others are optional). Maria is notoriously late in doing her readings, usually doing the minimum. Her friend Ariadne asks the day before class:
  1. Ariadne:         Dhiavases toulaxiston to ypoxreotiko arthro?
  2.                     “Did you read at least the required article?”
  3. Maria: a.         Ax, oxi! Dhen dhiavasa KANENA arthro!
  4.                       Ah, no! not read.1sg NPI.det article
  5.                 b.      Ax, oxi! #Dhen dhiavasa kanena arthro!
  6.                     “I didn't read any article at all!”

The non-emphatic NPI in (37a), in contrast to the emphatic one in (37b), is infelicitous. By using the *at-least* phrase in the question, the question forces a scalar reading (the required article is the most likely to read, or the least likely to ignore). The non-emphatic NPI is an odd device in the scalar context, while the emphatic NPI is fine. It is useful to see the parallel with *any*: *any* with devices such as *at all* differs from bare *any*, which can be used in statements that are rather neutral (see Duffley and Larrivée, 2012 for recent discussion).

(38) If you find any typos in this text, please let us know.
(39) Hitting any key will reactivate the screen.

Duffley and Larrivée (2012, p. 30) conclude that: “a good number of common uses of *any* are not amenable to a scalar interpretation at all,” as we can see in the examples above. In Greek NPIs, the *at all* intensification happens purely prosodically.

The prosodic distinction remained a theoretical generalization for many years since Veloudis' and Giannakidou's initial observations—but Chatzikonstantinou (2016) conducted production and comprehension experiments suggesting that the difference is observed empirically. For the purpose of this paper, we consider one of his production experiments. 30 native speakers (15 male, 15 female) of Greek were recruited aged from 22 to 55 (mean age 32). All of them were born in Greece and had completed at least the 12 obligatory years of school education while some of them had a higher education degree. The task was administered individually to each participant on a computer screen, and included slides with the scalar (such as (37) above) or non-scalar contexts. Each slide consisted of the context and a target sentence in bold font. The sentences were written in the Greek alphabet, and the whole information fitted in one slide. Each participant was instructed to first read the context, try to get a good understanding of it. There was no time limitation for this and participants were told that they can read the text as many times as they want. Upon this, the instructions guided them to read the target sentence aloud as if it was a kind of summary or continuation of what has been narrated in the context. Participants were also informed that during the whole process that their voice would be audio recorded. In each session, the experimenter was present and assisted with the procedure.

The scalar and non-scalar contexts presented the images we see in Graphemes 1, 2 (from Chatzikonstantinou, 2016). The contours are different, the most notable difference being the Low plateau within which the non-scalar NPI is realized. There is a High peak in the beginning of the utterance and then a deaccentuation till the end. The alignment of the High peak—here aligned with the S in the beginning of the utterance—varied as often it was aligned with the negative marker bearing a more typical negative contour. No intermediate phrase was observed as no pauses during the utterance were perceivable.

**Grapheme 1:**
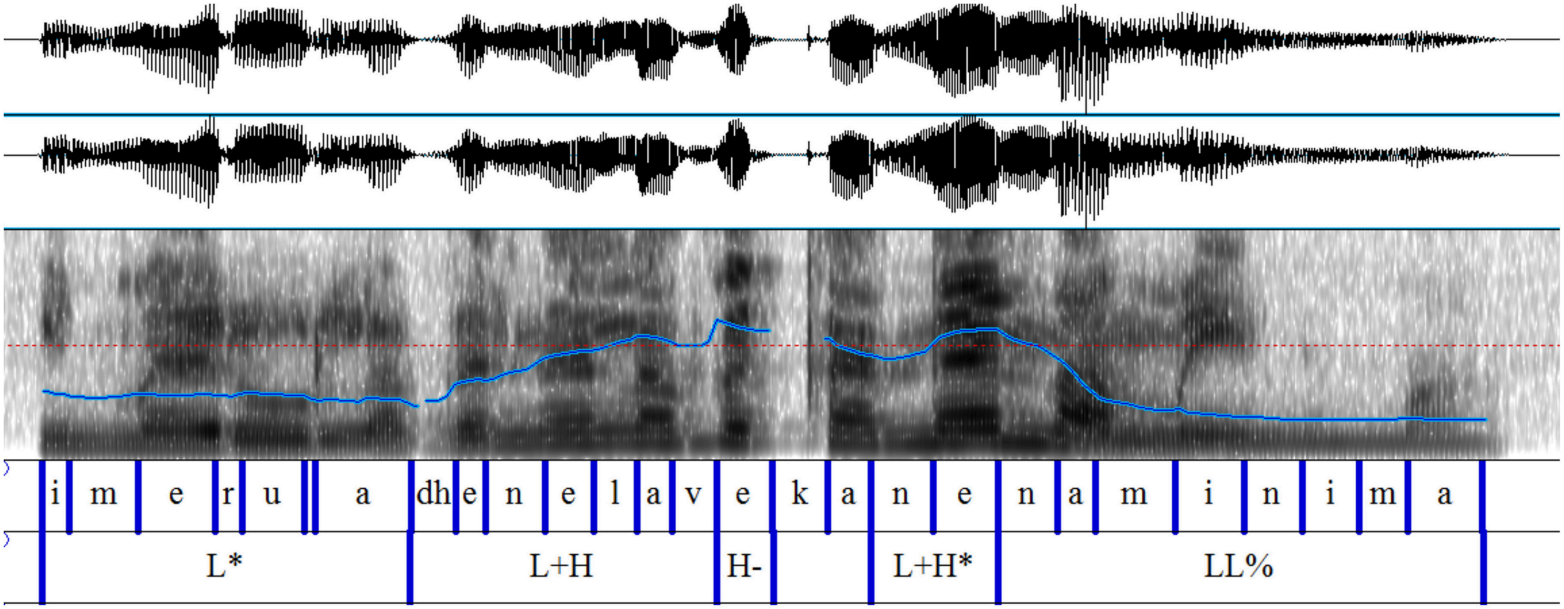
Sentential contour after a scalar context: exemplar waveform and spectrogram of scalar context with superimposed F0-contours.

**Grapheme 2:**
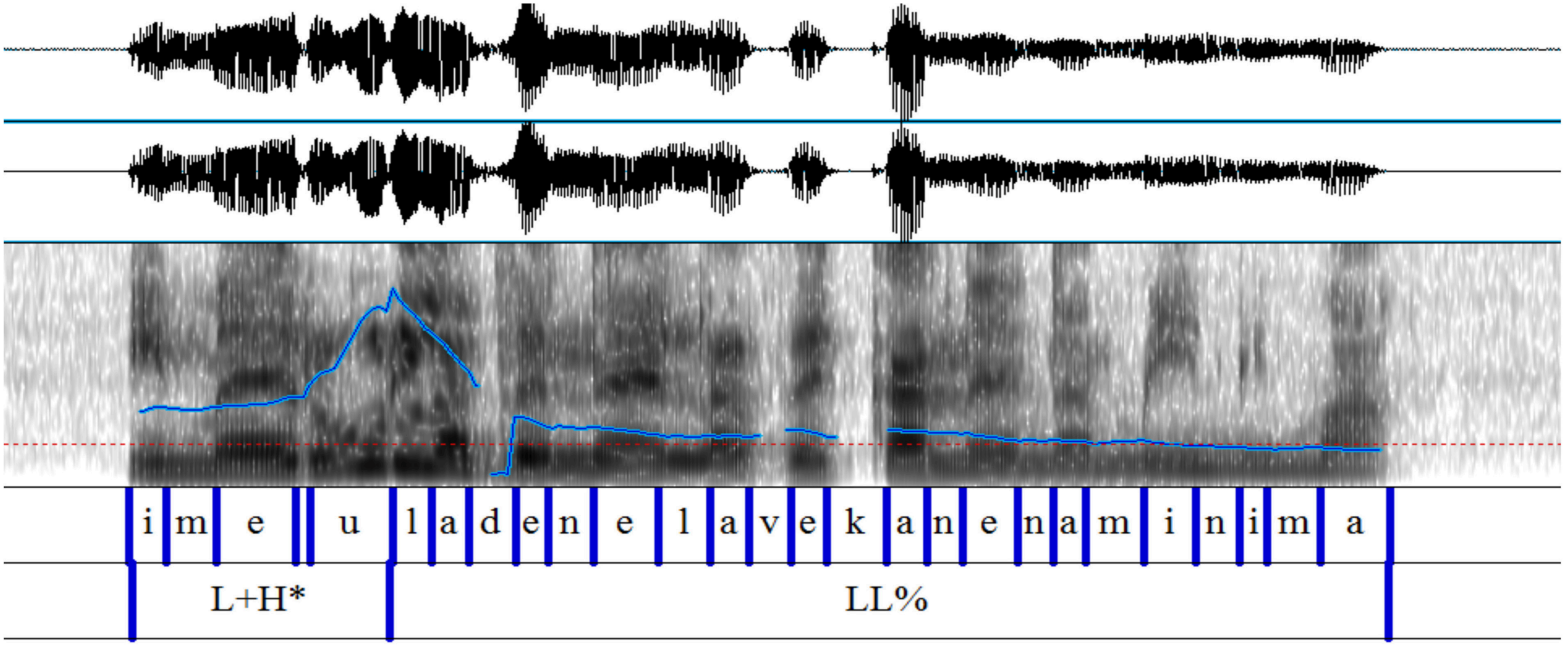
Sentential contour after a non-scalar context: exemplar waveform and spectrogram of non-scalar context with superimposed F0-contours.

Sentential contours are distinct in the two paradigms. The pitch contour (F-0) looks quite different: the emphatic is associated with a L+H^*^ (the H^*^ is aligned with the stressed syllable) and then a fall—but the non-emphatic has a flat intonation (and also the part before and after it).

Chatzikonstantinou investigated further the F0 and run a two way Anova (Scalarity (scalar, non-scalar) x Tonal Target (/e/ and /n/) contained in kan**e**nas [the bolded characters are the *tonal targets*)]. There was a significant main effect of *scalarity* on the pitch value produced, *F*_(1, 83)_ = 104,097, *p* < 0.001. There was also a main effect of *tonal target F*_(1, 83)_ = 18,859, *p* < 0.001 and an interaction effect between *scalarity* and *tonal target F* = 17.917, *p* < 0.001. The result suggests that Pitch is a robust acoustic cue that differentiates between a scalar and a non-scalar NPI.

Finally, duration measures were taken from /e/ and /n/ again and ran a two way Anova. The results show that there was a significant main effect of *scalarity* on the duration *F*_(1, 83)_ = 51,283, *p* < 0.001 which suggests that it makes a difference whether you are a scalar or a non-scalar NPI. A marginally significant effect on tonal target was also found [*F*_(1, 83)_ = 3,964, *p* < 0.05]. There was no interaction between the two factors (*F* = 1,621, *p* = 207).

To sum up, NPIs can be scalar and non-scalar, and the difference surfaces in prosodic properties; for more extensive discussion see Chatzikonstantinou's thesis, chapter 3. The important conclusion here is that the theoretical postulate of two prosodic profiles for Greek NPIs, which has been a mere theoretical statement for about 30 years, is actually confirmed by experimental data. This carries significant promise as one further explores the syntax-semantics and prosody interaction. We find next a similar pattern about the role of prosody in bringing about scalar and non-scalar focus in Basque.

### The basque additive particle

In Basque, the ordinary additive particle, *ere*, is used to express both a simple additive, non-scalar value (akin to English *too/also*) and a scalar additive value (akin to English *even*). In fact, *ere* is the only particle available in Basque to produce either simple additives or scalar additives (as opposed to other languages that have different items in the lexicon for different readings, e.g., *too/also* and *even* in English, *también* and *incluso* in Spanish, *aussi* and *meme* in French, etc.). Thus, in Basque, a string like (40), with the same lexical items and the same word order can obtain either a simple additive reading or a scalar additive reading:

(40) Mikel ere joan da.
Mikel ere go aux
*Simple:* Mikel left too.
*Scalar:* Even Mikel left.

At a first look, it would seem then that sentences containing the particle *ere* are completely ambiguous between the simple and the scalar additive interpretations. However, Etxeberria and Irurtzun (2015) show that prosody (the placement of the nuclear stress) is the key factor for teasing apart the two readings in (40). In order to verify the effect that prosody plays in disambiguating the simple and the scalar additive readings of *ere*, Etxeberria and Irurtzun designed two experiments: (i) a production experiment which aimed to test the prosodic patterns associated to each of the readings and, (ii) a perception experiment, a sentence-comprehension task where subjects had to judge the possible readings of utterances with the additive particle with varying prosodic patterns.

In the production experiment, they asked native speakers of Basque to utter pairs of identical strings corresponding to simple additive and scalar additive interpretations after presenting them a context (via written text) that clearly favored one of the two possible interpretations. They made use of three different strings, and two conditions per string, “Simple” and “Scalar,” and all of them contained the same syllable in the accented positions in the element preceding the particle *ere* (/ru/) and the verb following it (/di/). All participants read the same set of sentences. One of the strings they used is exemplified below between brackets “< >.”

**Figure 2:**
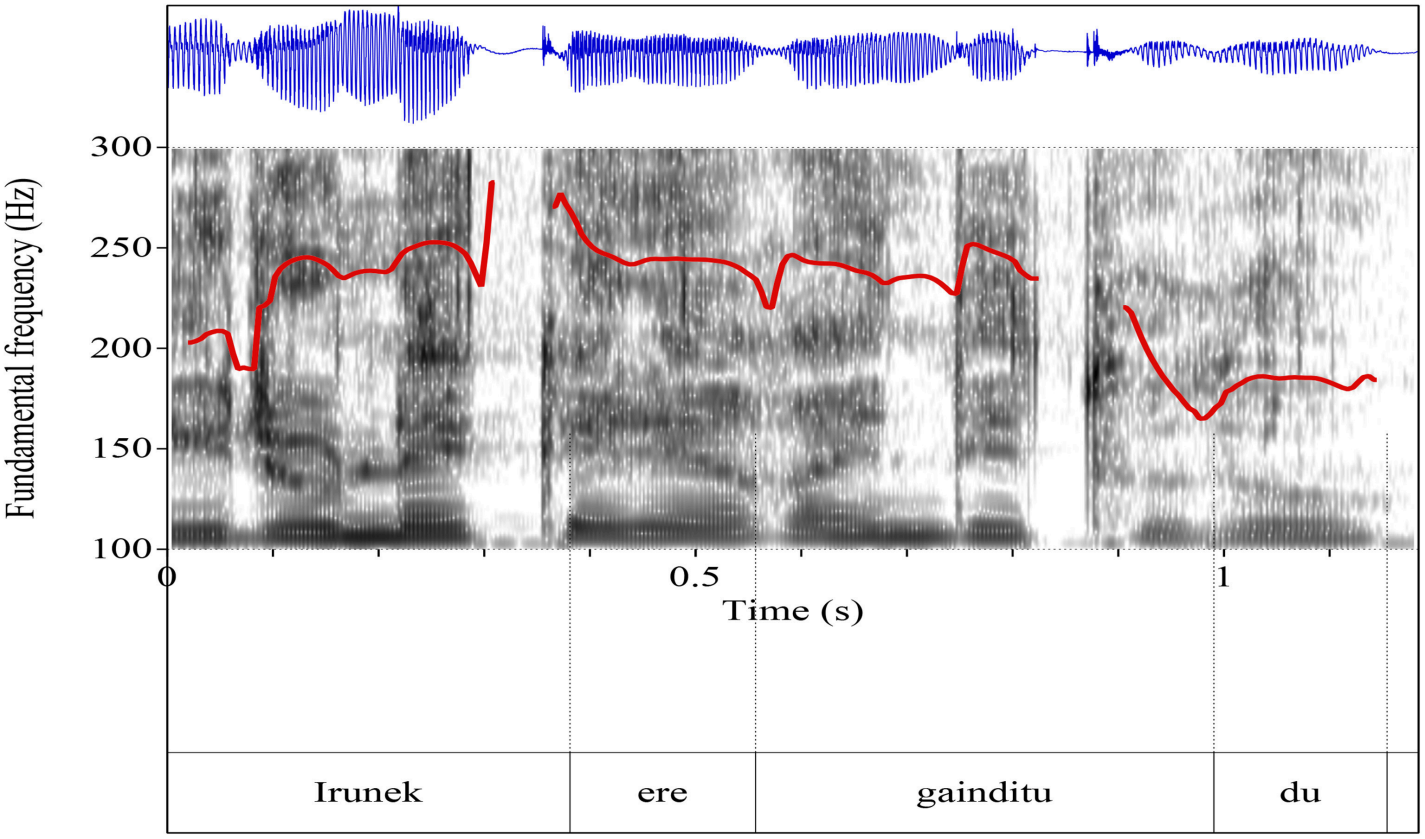
Pitch track of simple additive (*Irunek ere gainditu du* “Irune also passed it” or “Even Irune passed it”).

**Figure 3:**
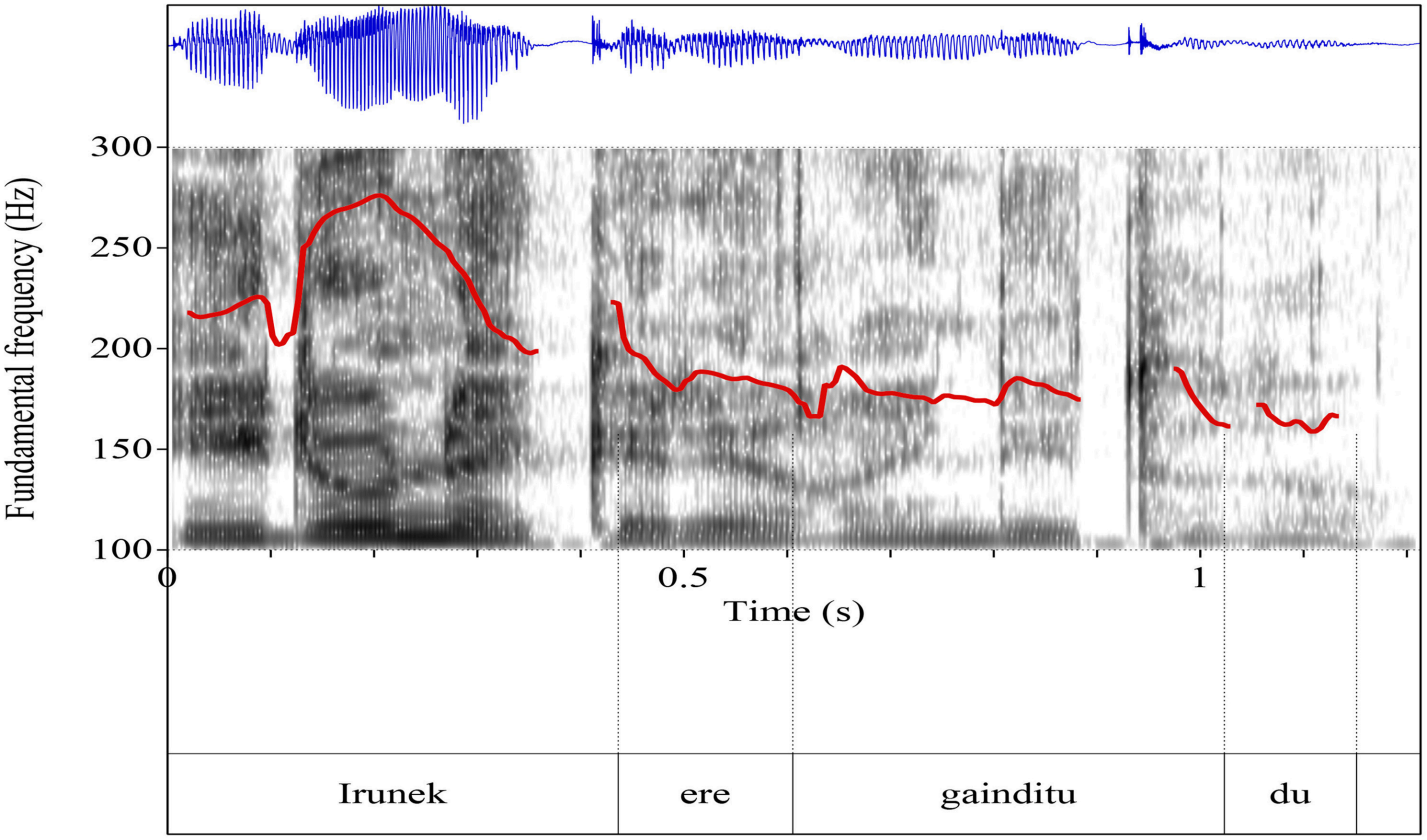
Pitch track of scalar additive (*Irunek ere gainditu du* “Irune also passed it” or “Even Irune passed it”).

(41)    a.    Simple (Figure 2):
                  Mertxek azterketa gaindittu do, eta <*Irunek ere*
                  *gaindittu do*>.
                  *English translation:* Mertxe passed the exam, and
                  <Irune ere (= too) passed the exam>.
          b.      Scalar (Figure 3):
                  Irune klaseko txarrena da, askokatik gainea. Askotan pasatzen da klaseko danok azterketetan nota ona ateatzea eta beak suspenditzea. Halare, lehengon jarri ziguten azterketa hain erraza izan zan, <*Irunek ere gaindittu dola*>.
                  *English translation:* Irune is, by far, the weakest in our class. Often times, we all get good grades and she gets an F. However, the exam that we got the other day was such an easy one that <Irune ere (= even) passed the exam>.

They measured syllable duration (in ms.), F0 mean and maxima (in Hertz), and intensity mean and maxima (in dB.) in the three syllables, as well as the F0 declination between F0 maxima in syllables /ru/ and /di/. The measurements show a clear difference between strings uttered in the simple condition and strings uttered in the scalar condition in that the stress associated to the element preceding the particle *ere* in the scalar condition is stronger (in F0 and intensity) than in the simple condition and they argue that this is a signature of their focal nature, since narrow focus is associated to nuclear stress in Basque.

Furthermore, they also show that in the Scalar condition the region following *ere* displays reduced F0 values in comparison to the Simple condition, which they linked to the well attested effect of postfocal pitch compression (cf. Elordieta, 1997, 2003; Elordieta and Irurtzun, 2009; Irurtzun, 2013; Hualde and Elordieta, 2014). They conclude that speakers associate different prosodic patterns to different interpretations of the same string, which is a remarkable fact because despite the fact that the contexts of utterance were unambiguous enough so that speakers would not convey any differences in their prosodic marking, i.e., despite the fact that the exact interpretation of *ere* (scalar and non-scalar) could be inferred from the context alone, speakers produce different tunes.

**Figure 4:**
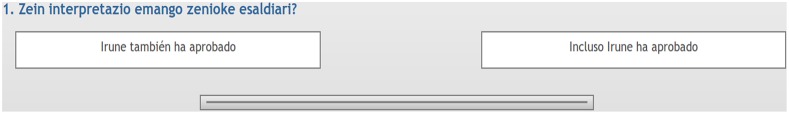
Visual analogue scale with unambiguous Spanish sentences at both ends (non-scalar simple additive *también* “also” on the left, *Irune también ha aprobado* “Irune also passed it”; scalar additive *incluso* “even” on the right, *Includo Irune ha aprobado* “Even Irune passed it”).

In order to check whether this intonational pattern is enough to convey the intended meaning, they run a perception experiment. For the perception experiment they designed a magnitude-estimation task with the help of a Visual Analogue Scale (VAS) with unambiguous interpretations at both ends (since all Central Basque speakers are bilingual speakers of Spanish and Basque, unambiguous Spanish sentences at both ends were used (with *también* “also”–*Irune también ha aprobado* “Irune also passed the exam”– and *incluso* “even” –*Incluso Irune ha aprobado* “Even Irune passed the exam”–) (Figure 4).

**Figure 5:**
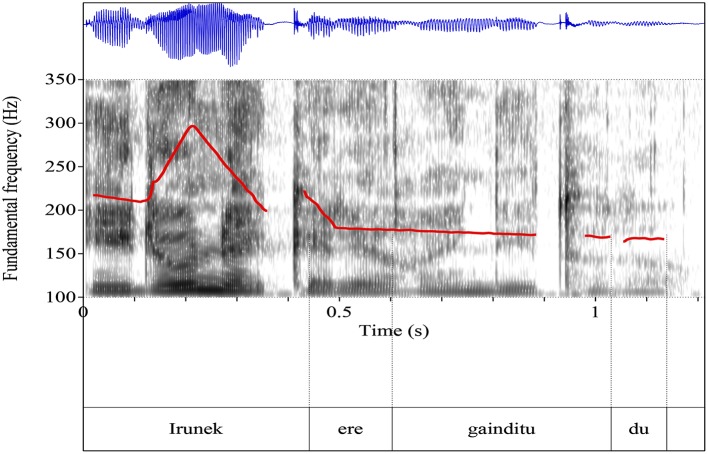
Condition Synth1 (*Irunek ere gainditu du* “Irune also passed it” or “Even Irune passed it”).

**Figure 6:**
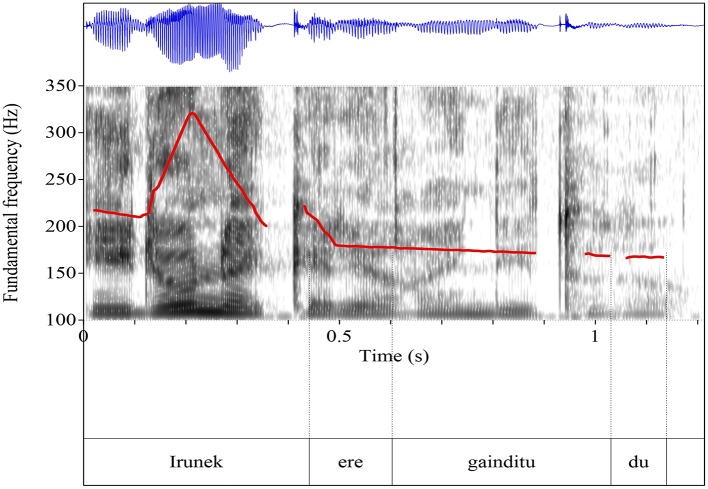
Condition Synth2 (*Irunek ere gainditu du* “Irune also passed it” or “Even Irune passed it”).

Participants had to listen to three strings uttered with two different interpretations (simple and additive) in mind, which were taken from the productions of a participant in production experiment. Besides, for the item *Irunek ere gainditu du* “(Even) Irune (too) passed the exam,” they created an additional pair of test items: Condition Synth1, a manipulation of the item for “Scalar” by stylizing F0, raising the peak of the pitch accent in the subject by 25 Hz, and flattening the postaccentual region (Figure 5), and Condition Synth2, a manipulation of the item for “Scalar” by stylizing F0, raising the peak of the pitch accent in the subject by 50 Hz and flattening the postaccentual region (Figure 6).

These experimental items (the same string with the same lexical items and the same word order in all cases, i.e., *Irunek ere gainditu du* “Irune also passed it” or “Even Irune passed it”) were offered to the participants without any kind of context and participants had to judge the range of possible interpretations of each utterance in the VAS by cutting the judgment line in two: (i) if they thought that the utterance was ambiguous and that it could equally represent the two readings, subjects were instructed to place the delimiter in the middle of the line; (ii) if they thought that it represented more the reading to the left, but still leaving some plausibility to the reading to the right they should place the delimiter on whichever place they felt on the left; (iii) alternatively, if they judged that the utterance was unambiguous in the other direction, they should place the delimiter more to the right. Subjects were explicitly instructed that they could place the delimiter at any point in the line. Etxeberria and Irurtzun controlled the validity of the technique with completely unambiguous fillers that could only be given one interpretation and hence should be placed at the extreme left or right boundary of the line.

**Figure 7:**
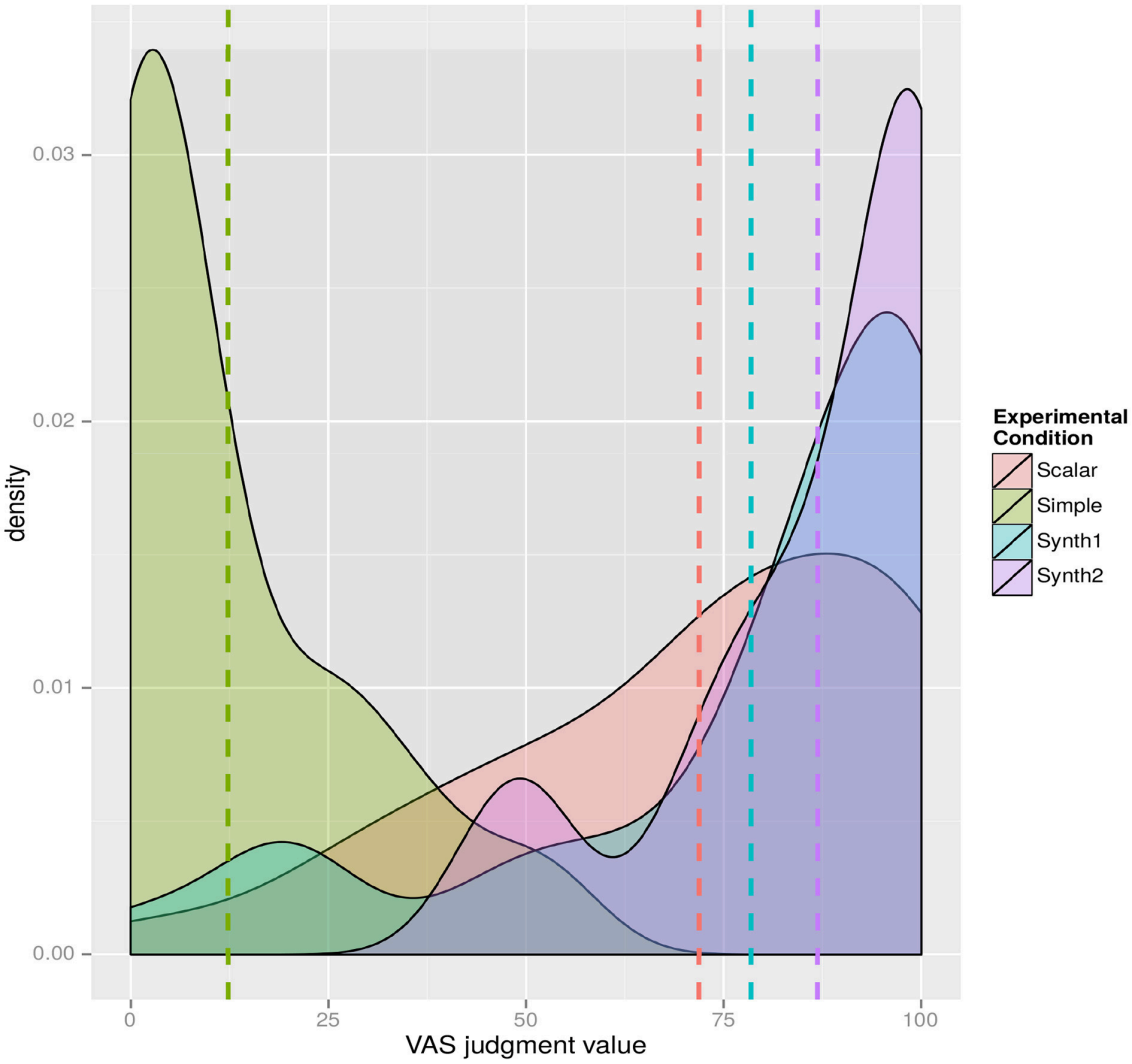
Density plot for judgments.

The results of the VAS (from 0 to 100, 0, the value on the leftmost edge, 100 the value on the rightmost edge) show a clearly skewed distribution (Simple *M* = 12.31, *SD* = 15.58; Scalar *M* = 71.88, *SD* = 26.37). The results are very interesting in that the stronger the accent the interpretation gets more scalar (Synth1 *M* = 78.47, *SD* = 28.74 and Synth2 *M* = 86.88, *SD* = 17.30). As Figure 6 shows, responses to different conditions show a different behavior, with clearly skewed distributions, significantly so in the cases of conditions Simple, Synth1 and Synth2 (Figure 7).

Thus, the paper by Etxeberria and Irurtzun shows that constructions with *ere* can vary in their interpretations between the simple and the scalar additivity readings but that these two readings differ depending on where the focal intonation, i.e., the nuclear stress, is placed. As a consequence, the two interpretations that can be obtained in Basque in sentences with *ere* are not to be considered as genuine ambiguity. In other words, there is a clear correspondence between the nonfocal or focal nature of the element preceding the additive particle *ere* and interpreting *ere* the sentence as simple additive or scalar additive. This shows that Basque make use of prosodic properties to disambiguate the scalar or non-scalar interpretations of the additive particle *ere*.

## General conclusions

Our goal in this article was to discuss one of the major questions addressed in this volume, namely if experimental methodologies are helpful in assessing linguistic data and theories about them. We reviewed some recent key literature on the licensing of negative polarity items (NPIs) and on the prosody-semantics interface. We found indeed that experimental methodologies allow us to establish and disentangle patterns and physical correlations of linguistic intuition that would otherwise remain undetected. The phenomena we reviewed involve what we called interface judgment, which is the intuition produced by integrating multiple levels of linguistic representation. We addressed three main areas of integration involving syntax, semantics and prosody.

ERP methodology, in particular, by tracking processing in time, was useful in establishing physical, quantitative correlates of NPI licensing. Reduced N400, we suggested, can be understood as the physical correlate of semantic licensing, and the observed P600 is an integration effect. In section Empirical Variation and the Scale of Negativity we saw that a mere acceptability judgment task was useful in revealing more sharpened intuitions about degrees of strength of NPI-licensers.

We chose NPIs and the focus particle EVEN because these are areas that we have studied in our previous works, and in the article we synthesized among results that included our own. Overall, the experimental methodologies allowed us to tease apart the key aspects of grammatical judgment with NPI licensing, including prosodic properties of NPIs. In addition, disambiguation of scalar and non-scalar readings of a single word (Greek NPI, Basque *ere*) was clearly established with the aid of phonological experimental observation. Our overall conclusion is that we can be hopeful that experimental methodology can be a helpful tool for interface judgment in revealing the actual empirical patterns that are relevant for theorizing.

## Author contributions

All authors listed have made a substantial, direct and intellectual contribution to the work, and approved it for publication.

### Conflict of interest statement

The authors declare that the research was conducted in the absence of any commercial or financial relationships that could be construed as a potential conflict of interest.

## Footnotes

^1^Wellwood et al. (under review) discuss a similar malleability observed in comparative constructions like *More people have been to Russia than I have*. Such sentences tend to be perceived as meaningful by native speakers of English, but upon closer reflection, they are judged to be incoherent.

^2^Crucially, it is not our goal to offer a comprehensive account of what might be meant by “integration” in terms of a finite set (or classes) of operations. The syntax semantics interface, for instance, and the prosody semantics interface will involve rather different integration operations—and some methodologies will likewise be better suited to capture certain integrations than others. It is definitely desirable to reach an account of operations that sets these apart in some interesting way from other kinds of processing, but that would require more extensive consideration of data than we can offer at present. We thank John Drury for raising this question.

^3^*Any* can receive free choice readings in modal contexts (Giannakidou, 2001; Giannakidou and Cheng, 2006). We omit consideration of free choice here to keep things simple. Free choice is also a polarity phenomenon.

^4^Nonveridicality plays a decisive role in the selection of subjunctive and other non-assertive moods (see Giannakidou, 1995, 1998, 2009; Quer, 1998), hence it is a logical category with broader applications in grammar. Mood selection can certainly be understood as an interface phenomenon.

^5^NPIs are often discussed in contradistinction to positive polarity items (PPIs). Often a duality is imposed (Progovac, 2005), but Giannakidou (2011, 2017) cautions that the PPI failures are not of the same kind:

(i) a. John unfortunately died
   b. John didn't unfortunately die.

Metalinguistic denial (Horn, 1989) can rectify PPIs (see Ernst, 2009): *John isn't here ALREADY, we are still waiting for him;* hence “#” reflects that the PPI is not ungrammatical, but infelicitous in the scope of negation. A failed NPI is ungrammatical, but a failed PPI appears to be only infelicitous, the intended duality between NPIs and PPIs thus does not involve necessarily the same grammatical mechanism. Krifka (1995) suggests a Gricean explanation that since under negation the NPI is the default, using another form implicates that the alternative scope is intended. Experimental evidence can be useful in distinguishing between NPIs and PPIs, but this will be a topic for future research.

^6^The concept of dependent variable itself does not necessitate the POL feature. A failed dependent variable can remain a pure presupposition failure. E.g., the presupposition of #*He left* is not satisfied in a female-only domain, but this does not cause ungrammaticality. Dependent variable phenomena are not *all* NPI phenomena (e.g., distributivity markers or controlled subjects are not NPIs); and some polarity phenomena might be purely presupposition failures, as Giannakidou accepts in numerous places (e.g., PPIs, as suggested earlier).

^7^Multiple NPIs were tested in Steinhauer et al. (2010). N400 difference was only observed for *at all*, but not for *ever* or *any*. Let us also mention that the NPI studies did not always examine NPI licensing alone. To give some examples, Saddy et al. (2004) and Yurchenko et al. (2013) compared PPI and NPI licensing contexts within the same experiment, Xiang et al. (2009) compared NPI licensing with reflexives and some of the German and English studies included an inaccessible licensor condition besides the no-licensor vs. licensor distinction in their manipulation. All these things matter for the possible elicitation of different ERP components.

^8^Only two studies did not find a P600: Saddy et al. (2004) and Yurchenko et al. (2013). The original data from Saddy et al. were reanalyzed in Drenhaus et al. (2006) using a symbolic resonance analysis, and a hidden P600 was discovered. Yurchenko et al. acknowledged that the lack of a P600 may be due to insufficient power in the data, as well as, potentially, to task-specific effects.

^9^Importantly, we do not imply seriality in processing stages as e.g., in the three-phase model of Friederici (1995, 2002), Friederici and Kotz (2003), Friederici and Weissenborn (2007). Steinhauer and Drury offer a critique of that model, concluding that “the three-stage architecture of Friederici's model may have to be modified as there seems to be little evidence for a first phase exclusively dedicated to phrase structure processing. Moreover, context-driven top-down processing may play a larger role than assumed by the current version of this model.” (Steinhauer and Drury, 2012, p. 154). Our point in this paper is simply to raise awareness that N400 and P600 are physical correlates of licensing; we remain agnostic wrt to architecture that clearly requires more study.

^10^It is worth mentioning here the so-called “semantic P600,” which sometimes appears with an absence of N400 effect. Chow and Phillips (2013) offer a detailed discussion arguing that semantic P600 is compatible with the long-held assumption that online semantic composition is dependent on surface syntax.

## References

[B1] Aloni M. (2007). Free choice, modals and imperatives. Nat. Lang. Semantics 15, 65–94. 10.1007/s11050-007-9010-2

[B2] Baker C. L. (1970). Double negatives. Linguist. Inq. 1, 169–186.

[B3] Beaver D. (2010), Have you noticed that your belly button lint colour is related to the colour of your clothing?, in Presuppositions and Discourse: Essays Offered to Hans Kamp, eds Bäuerle R. Reyle U. Zimmerman T. E. (Bingley: Emerald Group Publishing Limited), 65–100.

[B4] Bornkessel-Schlesewsky I. Schlesewsky M. (2008). An alternative perspective on “semantic P600” effects in language comprehension. Brain Res. Rev. 59, 55–73. 10.1016/j.brainresrev.2008.05.003 18617270

[B5] Büring D. (1997). The great scope inversion conspiracy. Linguist. Philos. 20, 175–194. 10.1023/A:1005397026866

[B6] Chatzikonstantinou A. (2016). Semantic and Prosodic Processing of Negative Polarity Items in Greek. Ph.D. University of Chicago, Chicago, IL.

[B7] Chatzikontantinou A. Giannakidou A. Manouilidou C. (2015). Gradient strength of NPI-licensers in Greek, in Paper Presented at the 12th International Conference on Greek Linguistics (Potsdam: University of Potsdam).

[B8] Chierchia G. (2006). Broaden your views. Implicatures of domain widening and the ‘logicality’ of language. Linguist. Inq. 37, 535–590. 10.1162/ling.2006.37.4.535

[B9] Chierchia G. (2013). Logic in Grammar. Polarity, Free Choice, and Intervention. Oxford: Oxford University Press.

[B10] Chomsky N. (1957). Syntactic Structures. The Hague: Mouton.

[B11] Chomsky N. (1964). Current Issues in Linguistic Theory. The Hague: Mouton.

[B12] Chomsky N. (1981). Lectures on Government and Binding. Dordrecht: Foris Publications.

[B13] Chow W.-Y. Phillips C. (2013). No semantic illusion in the “Semantic P600” phenomenon: ERP evidence from Mandarin Chinese. Brain Res. 1506, 76–93. 10.1016/j.brainres.2013.02.016 23422676

[B14] Drenhaus H. Beim Graben P. Saddy D. Frisch S. (2006). Diagnosis and repair of negative polarity constructions in the light of symbolic resonance analysis. Brain Lang. 96, 255–268. 10.1016/j.bandl.2005.05.001 15975647

[B15] Drenhaus H. Blaszczak J. Schutte J. (2007). Some psycholinguistic commentson NPI licensing, in Proceedings of Sinn und Bedeutung, Vol. 11, ed Puig-Waldmuller E. (Barcelona: Universitat Pompeu Fabra), 180–193.

[B16] Drenhaus H. Saddy D. Frisch S. (2005). Processing negative polarity items: When negation comes through the backdoor, in Linguistic Evidence—Empirical, Theoretical, and Computational Perspectives, eds Kepser S. Reis M. (Berlin; New York, NY: Mouton de Gruyter), 145–165.

[B17] Duffley P. Larrivée P. (2012). Collocation, interpretation and explanation: the case of JUST ANY. Lingua 122, 24–40. 10.1016/j.lingua.2011.10.008

[B18] Duffley P. Larrivée P. (2015). A fresh look at the incompatibility between *any* and veridical contexts. The quality of indefiniteness is not strained. Lingua 158, 35–53. 10.1016/j.lingua.2015.01.004

[B19] Elordieta G. (1997). Accent, tone and intonation in lekeitio basque, in Issues in the Phonology and Morphology of the Major Iberian Lanuages, eds Fernando M.-G. Alfonso M.-F. (Washington, DC: Georgetown University Press), 4–78.

[B20] Elordieta G. (2003). Intonation, in A Grammar of Basque, eds Hualde J. I. Ortiz de Urbina J. (Berlin: Mouton de Gruyter), 72–112.

[B21] Elordieta G. Irurtzun A. (2009). The relationship between meaning and intonation in nonexhaustive answers: evidence from Basque. Linguist. Rev. 3, 261–291.

[B22] Ernst T. (2009). Speaker oriented adverbs. Nat. Lang. Linguist. Theory 27, 497–544. 10.1007/s11049-009-9069-1

[B23] Etxeberria U. Irurtzun A. (2004). Prosodic features with semantic interpretation, in Proceedings of JEL 2004 Domaines, eds Demirdache H. Waquier-Graveline S. Crouzet O. (Nantes: AAI), 95–101.

[B24] Etxeberria U. Irurtzun A. (2015). The emergence of scalar meanings. Front. Psychol. 6:141. 10.3389/fpsyg.2015.00141 25745405 PMC4333776

[B25] Farkas D. (1998). Dependent indefinites, in Empirical Issues in Formal Syntax and Semantics, eds Corblin F. Godard D. Marandin J.-M. (Bern: Peter Lang Publishers), 243–268.

[B26] Fauconnier G. (1975). Polarity and the Scale Principle, Vol. 11. Chicago, IL: Chicago Linguistics Society.

[B27] Friederici A. D. (1995). The time course of syntactic activation during language processing: a model based on neuropsychological and neurophysiological data. Brain Lang. 50, 259–281. 10.1006/brln.1995.1048 7583190

[B28] Friederici A. D. (2002). Towards a neural basis of auditory sentence processing. Trends Cogn. Sci. 6, 78–84. 10.1016/S1364-6613(00)01839-8 15866191

[B29] Friederici A. D. Kotz S. A. (2003). The brain basis of syntactic processes: functional imaging and lesion studies. Neuroimage 20, S8–S17. 10.1016/j.neuroimage.2003.09.003 14597292

[B30] Friederici A. D. Weissenborn J. (2007). Mapping sentence form onto meaning: the syntax-semantic interface. Brain Res. 1146, 50–58. 10.1016/j.brainres.2006.08.038 16956590

[B31] Gajewski J. (2002). L-analyticity and Natural Language, Cambridge, MA: MIT.

[B32] Giannakidou A. (1995). Linking mood to polarity sensitivity: the case of the Modern Greek subjunctive, in Proceedings of CONSOLE 3, eds Bisetti A. Costa J. Goedemans R. Munaro N. van de Vijver R. (Venice: University of Venice), 71–83.

[B33] Giannakidou A. (1997). The Landscape of Polarity Items. Ph.D, University of Groningen.

[B34] Giannakidou A. (1998). Polarity Sensitivity as (non)Veridical Dependency. Amsterdam: John Benjamins.

[B35] Giannakidou A. (2000). Negative …concord? Nat. Lang. Linguist. Theory 18, 457–523. 10.1023/A:1006477315705

[B36] Giannakidou A. (2001). The meaning of free choice. Linguist. Philos. 24, 659–735. 10.1023/A:1012758115458

[B37] Giannakidou A. (2006). Only, emotive factives, and the dual nature of polarity dependency. Language (Baltim) 82, 575–603. 10.1353/lan.2006.0136

[B38] Giannakidou A. (2009). The dependency of the subjunctive revisited: temporal semantics and polarity. Lingua 119, 1883–1908. 10.1016/j.lingua.2008.11.007

[B39] Giannakidou A. (2011). Negative and positive polarity items, in Semantics: An International Handbook of Natural Language Meaning, eds von Heusinger K. Maienborn C. Portner P. (Berlin: de Gruyter), 1660–1712.

[B40] Giannakidou A. (2016). Evaluative subjunctive and non-verididicality, in Revisiting Mood, Aspect and Modality: What is a Linguistic Category, eds Blaszczak J. Anastasia Giannakidou D. Klimek-Jankowska K. (University of Chicago Press), 141–173. 10.7208/chicago/9780226363660.003.0005

[B41] Giannakidou A. (2017). Polarity in the Semantics of Natural Language. Oxford Encyclopedia in Linguistics. Oxford: Oxford University Press.

[B42] Giannakidou A. Cheng L. (2006). (In)definiteness, polarity, and the role of wh-morphology in free choice. J. Semant. 23, 135–183. 10.1093/jos/ffl001

[B43] Giannakidou A. Mari A. (2018). A unified analysis of the future as epistemic modality: the view from Greek and Italian. Nat. Lan. Linguist. Theory. 36, 85–129. 10.1007/s11049-017-9366-z

[B44] Giannakidou A. Quer J. (2013). Exhaustive and non-exhaustive variation with anti-specific indefinites: free choice and referential vagueness in Greek, Catalan, and Spanish. Lingua 26, 120–149. 10.1016/j.lingua.2012.12.005

[B45] Giannakidou A. Yoon S. (2016). Scalar marking without scalar meaning: non-scalar, non-exhaustive NPIs in Greek and Korean. Language (Baltim) 92, 522–556. 10.1353/lan.2016.0047

[B46] Giannakidou A. Zeijlstra H. (2017). The landscape of negative dependencies: negative concord and n-words, in The Blackwell Companion to Syntax, 2nd Edn., Eds Evaerert M. van Riemsdijk H. 10.1002/9781118358733.wbsyncom10

[B47] Gouvêa A. C. Phillips C. Kazanina N. Poeppel D. (2010). The linguistic processes underlying the P600. Lang. Cogn. Process. 25, 149–188. 10.1080/01690960902965951

[B48] Grano T. (2011). Mental action and event structure in the semantics of try, in Semantics and Linguistic Theory, eds Chereches A. Lutz D. (New Brunswick, NJ: Rutgers University), 426–443.

[B49] Hagoort P. Brown C. M. Groothusen J. (1993). The syntactic positive shift as an ERP measure of syntactic processing. Lang. Cogn. Process. 8, 439–483.

[B50] Hoeksema J. (2010). Dutch *ENIG*: from nonveridicality to downward entailment. Nat. Lang. Linguist. Theory 28, 837–859. 10.1007/s11049-010-9110-4

[B51] Horn L. (1972). On the Semantic Properties of Logical Operators in English, Ph.D. UCLA.

[B52] Horn L. (1989). A Natural History of Negation. Stanford, CA: CSLI Publications.

[B53] Horn L. (2002). Assertoric inertia and NPI licensing, in Proceedings of the Annual Meeting of the Chicago Linguistic Society (Chicago, IL University of Chicago), 38.

[B54] Hualde J. I. Elordieta G. (2014). Intonation in Basque, in Prosodic Typology II: The Phonology of Intonation and Phrasing, ed Jun S.-A. (Oxford: Oxford University Press), 405–463.

[B55] Irurtzun A. (2013). Focal NSR and stress placement in Basque phrases and N+N compounds. Linguist. Anal. 38, 207–242.

[B56] Israel M. (1996). Polarity sensitivity as lexical semantics. Linguist. Philos. 19, 619–666.

[B57] Israel M. (2011). The Grammar of Polarity: Pragmatics, Sensitivity, and the Logic of Scales. Cambridge: Cambridge University Press.

[B58] Jackendoff R. (1972). Semantic Interpretation in Generative Grammar. Cambridge, MA: MIT Press.

[B59] Jackson S. (2007). Information, Truth, Structure, and Sound. Ph.D, University of Arizona.

[B60] Kaan E. Harris A. Gibson E. Holcomb P. J. (2000). The P600 as an index of syntactic integration difficulty. Lang. Cogn. Process. 15, 159–201. 10.1080/016909600386084

[B61] Kadmon N. Landman F. (1993). ‘Any’. Linguist. Philos. 16, 353–422. 10.1007/BF00985272

[B62] Kennelly S. D. (2003). The implication of quantification for the role of focus in discourse structure. Lingua 113, 1005–1088. 10.1016/S0024-3841(03)00013-5

[B63] Kennelly S. D. (2004). Quantificational Dependencies. Ph.D. Dissertation, University of Utrecht.

[B64] Klima E. S. (1964). Negation in English, in The Structure of Language, eds Fodor J. A. Katz J. J. (Englewood Cliffs, NJ: Prentice-Hall), 246–323.

[B65] Krifka M. (1995). The semantics and pragmatics of polarity items in assertion. Linguist. Anal. 15, 209–257.

[B66] Krifka M. (1998). Scope inversion under the rise-fall contour in German. Linguist. Inq. 29, 75–112. 10.1162/002438998553662

[B67] Kuperberg G. R. (2007). Neural mechanisms of language comprehension: challenges to syntax. Brain Res. 1146, 23–49. 10.1016/j.brainres.2006.12.063 17400197

[B68] Kuperberg G. R. (2013). The pro-active comprehender: what event-related potentials tell us about the dynamics of reading comprehension, in Unraveling the Behavioral, Neurobiological, and Genetic Components of Reading Comprehension, eds Miller B. Cutting L. McCardle P. (Baltimore: Paul Brookes Publishing), 176–192.

[B69] Kutas M. Federmeier K. D. (2011). Thirty years and counting: finding meaning in the N400 component of the event-related brain potential (ERP). Annu. Rev. Psychol. 62, 621–647. 10.1146/annurev.psych.093008.131123 20809790 PMC4052444

[B70] Ladusaw W. A. (1980). Negative Polarity Items as Inherent Scope Relations. Ph.D, Dissertation, University of Texas at Austin.

[B71] Laka I. (1994). On the Syntax of Negation. Outstanding Dissertations in Linguistics Series, New York, NY; London: Garland Publishing Co.

[B72] Lau E. F. Phillips C. Poeppel D. (2008). A cortical network for semantics: [de]constructing the N400. Nat. Rev. Neurosci. 9, 920–933. 10.1038/nrn2532 19020511

[B73] Li F. González-Fuente S. Pietro P. Espinal M. T. (2016). Is mandarin chinese a truth-based language? Rejecting responses to negative assertions and questions. Front. Psychol. 7:1967. 10.3389/fpsyg.2016.01967 28066292 PMC5167746

[B74] Lin J. (2015). On the Acquisition of Negative Polarity Items. Ph.D. University of Amsterdam.

[B75] Linebarger M. (1987). Negative polarity and grammatical representation. Linguist. Philos. 10, 325–387. 10.1007/BF00584131

[B76] Martí L. (2001). Intonation, scope and restriction on quantifiers, in Proceedings of WCCFL 20, eds Megerdoomian K. Bar-el L. (Somersville, MA: Cascadilla Press), 372–385.

[B77] Menéndez-Benito P. (2010). On universal free choice items. Nat. Lang. Semant. 18, 33–64. 10.1007/s11050-009-9050-x

[B78] Milsark G. (1977). Toward an explanation of certain peculiarities of the existential construction in English. Linguis. Anal. 3, 1–28.

[B79] Osterhout L. Holcomb P. J. (1992). Event-related potentials elicited by syntactic anomaly. J. Mem. Lang. 31, 785–806. 10.1016/0749-596X(92)90039-Z

[B80] Osterhout L. Holcomb P. J. Swinney D. A. (1994). Brain potentials elicited by garden-path sentences: evidence of the application of verb information during parsing. J. Exp. Psychol. Learn. Mem. Cogn. 20, 786–803. 10.1037/0278-7393.20.4.786 8064247

[B81] Partee B. H. (1988). Many quantifiers, in Proceedings of Fifth ESCOL, eds Powers J. de Jong K. (Columbus, OH: The Ohio State University), 383–402.

[B82] Phillips C. Kazanina N. Abada S. (2005). ERP effects of the processing of syntactic long-distance dependencies. Cogn. Brain Res. 22, 407–428. 10.1016/j.cogbrainres.2004.09.012 15722211

[B83] Progovac L. (1994). Positive and Negative Polarity: A Binding Approach. Cambridge: Cambridge University Press.

[B84] Progovac L. (2005). Negative and positive feature checking and the distribution of polarity items, in Negation in Slavic, eds Brown S. Przepiorkowski A. (Bloomington, IN: Slavica Publishers), 179–217.

[B85] Quer J. (1998). Mood at the Interface. Ph.D. thesis. University of Utrecht.

[B86] Saddy D. Drenhaus H. Frisch S. (2004). Processing polarity items: contrasting licensing costs. Brain Lang. 90, 495–502. 10.1016/S0093-934X(03)00470-X 15172566

[B87] Shao J. Neville H. (1998). Analyzing semantic processing using event-related potentials. Newsl. Center Res. Lang. 11, 3–20.

[B88] Simons M. David B. Craige R. Judith T. (2017). The best question: explaining the projection behavior of factives. Discourse Process. 54, 187–206. 10.1080/0163853X.2016.1150660

[B89] Steinhauer K. Drury J. E. (2012). On the early left-anterior negativity (ELAN) in syntax studies. Brain Lang. 120, 135–162. 10.1016/j.bandl.2011.07.001 21924483

[B90] Steinhauer S. Drury J. Portner P. Walenski M. Ullman M. (2010). Syntax, concepts, and logic in the temporal dynamics of language comprehension: evidence from event-related potentials. Neuropsychologia, 48, 1525–1542. 10.1016/j.neuropsychologia.2010.01.013 20138065 PMC2862874

[B91] Tesan G. Johnson B. W. Crain S. (2012). How the brain responds to any: an MEG study. Brain Lang. 120, 66–72. 10.1016/j.bandl.2011.08.006 21944227

[B92] Tonhauser J. (2016). Prosodic cues to presupposition projection, in Semantics and Linguistic Theory (SALT), Vol. 26, eds Carol-Rose C. J. M. Little M. Burgdorf D. (Austin, TX: University of Texas), 934–960.

[B93] Tsimpli I. M. Roussou A. (1996). Negation and polarity items in Modern Greek. Linguist. Rev. 13, 1–33. 10.1515/tlir.1996.13.1.49

[B94] Van Petten C. Luka B. J. (2012). Prediction during language comprehension: benefits, costs, and ERP components. Int. J. Psychophysiol. 83, 176–190. 10.1016/j.ijpsycho.2011.09.015 22019481

[B95] Veloudis I. (1982). Negation in Modern Greek. Ph.D, University of Reading.

[B96] von Fintel K. (1999). NPI-licensing, strawson-entailment, and context-dependency. J. Semant. 16, 97–148. 10.1093/jos/16.2.97

[B97] Xiang M. Dillon B. Phillips C. (2009). Illusory licensing effects across dependency types: ERP evidence. Brain Lang. 108, 40–55. 10.1016/j.bandl.2008.10.002 19007980

[B98] Xiang M. Grove J. Giannakidou A. (2013). Dependency-dependent interference: NPI interference, agreement attraction, and global pragmatic inferences. Front. Psychol. 4:708. 10.3389/fpsyg.2013.00708 24109468 PMC3791380

[B99] Xiang M. Grove J. Giannakidou A. (2016). Semantic and pragmatic processes in the comprehension of negation: an event related potential study of negative polarity items. J. Neurolinguistics 38, 71–88. 10.1016/j.jneuroling.2015.11.001

[B100] Yanilmaz A. Drury J. E. (2018). Prospective NPI licensing and intrusion in Turkish. Lang. Cogn. Neurosci. 33, 111–138. 10.1080/23273798.2017.1371779

[B101] Yurchenko A. den Ouden D. B. Hoeksema J. Dragoy O. Hoeks J. Stowe L. (2013). Processing polarity: ERP evidence for differences between positive and negative polarity. Neuropsychologia 5, 132–141. 10.1016/j.neuropsychologia.2012.10.028 23142350

[B102] Zeijlstra H. (2004). Sentential Negation and Negative Concord. Ph.D, University of Amsterdam.

[B103] Zwarts F. (1995). Nonveridical con-texts. Linguist. Anal. 25, 286–312.

[B104] Zwarts F. (1996). A hierarchy of negative expressions in Negation: A Notion in Focus, ed Wansing H. (Berlin: de Gruyter), 169–194.

